# Understanding the Structure, Multimerization, Subcellular Localization and mC Selectivity of a Genomic Mutator and Anti-HIV Factor APOBEC3H

**DOI:** 10.1038/s41598-018-21955-0

**Published:** 2018-02-28

**Authors:** Fumiaki Ito, Hanjing Yang, Xiao Xiao, Shu-Xing Li, Aaron Wolfe, Brett Zirkle, Vagan Arutiunian, Xiaojiang S. Chen

**Affiliations:** 1 Molecular and Computational Biology, Departments of Biological Sciences and Chemistry, University of Southern California, Los Angeles, CA 90089 USA; 2 Genetic, Molecular and Cellular Biology Program, Keck School of Medicine, University of Southern California, Los Angeles, CA 90089 USA; 3 Center of Excellence in NanoBiophysics, University of Southern California, Los Angeles, CA 90089 USA; 4 Norris Comprehensive Cancer Center, University of Southern California, Los Angeles, CA 90089 USA; 5 Present Address: Department of Infectious Diseases and Vaccines Research, Merck Research Laboratories, Merck & Co., Inc, West Point, PA USA; 6 Present Address: Department of Internal Medicine, Meharry Medical College, Nashville, TN USA

## Abstract

APOBEC3H (A3H) is a member of the APOBEC3 subfamily of DNA cytosine deaminases that are important for innate immune defense and have been implicated in cancer biogenesis. To understand the structural basis for A3H biochemical function, we determined a high-resolution structure of human A3H and performed extensive biochemical analysis. The 2.49 Å crystal structure reveals a uniquely long C-terminal helix 6 (h6), a disrupted β5 strand of the canonical five-stranded β-sheet core, and a long loop 1 around the Zn-active center. Mutation of a loop 7 residue, W115, disrupted the RNA-mediated dimerization of A3H yielding an RNA-free monomeric form that still possessed nucleic acid binding and deaminase activity. A3H expressed in HEK293T cells showed RNA dependent HMW complex formation and RNase A-dependent deaminase activity. A3H has a highly positively charged surface surrounding the Zn-active center, and multiple positively charged residues within this charged surface play an important role in the RNA-mediated HMW formation and deaminase inhibition. Furthermore, these positively charged residues affect subcellular localization of A3H between the nucleus and cytosol. Finally, we have identified multiple residues of loop 1 and 7 that contribute to the overall deaminase activity and the methylcytosine selectivity.

## Introduction

APOBEC3H (A3H) is one of the APOBEC family members of DNA cytosine deaminases that play important roles in immune function (reviewed in^1–3^ and references therein), including restricting endogenous retroelements and infectious retroviruses^4–9^ and antibody maturation^10–13^. As the most divergent member of the A3 family, A3H belongs to the Z3-type Zn-coordination domain that is phylogenetically distinct from the Z1- and Z2-type domains of other A3 proteins^14,15^. A3H is the most polymorphic member of the APOBEC family^14,16,17^, as its mRNA can undergo alternative splicing to generate four splicing variants (SVs), and has seven distinct human A3H haplotypes (hap I-VII) containing various combinations of five single nucleotide polymorphisms^6,18,19^.

Among the seven A3H haplotypes, only hap II, V, and VII are reported to effectively restrict Vif-deficient HIV-1^6,18–21^, even though hap I was also shown to have anti-HIV activity when overexpressed in cell culture^18,19,22,23^. Literature suggests that the anti-HIV activity of A3H can be through both deaminase dependent and independent manners^18,24^, and the different anti-HIV activities of the A3H variants were attributed to a combination of different factors, such as differences in RNA binding and virion packaging^23,25^, protein stability^6,26–28^, deaminase activity^29^, and subcellular localization^22,23^.

Nucleic acid binding is a key feature of all APOBEC proteins, and nucleic acids can often have multiple roles in function and activity. A3H has been found in different oligomeric forms, and it can oligomerize both in cells and during recombinant protein purification^27,29–31^. Evidence so far suggests that binding to RNA is largely responsible for the multimerization of A3H and some other APOBEC members^24,30,32–35^. RNA binding is also an important step for the recruitment and encapsidation of APOBEC proteins to the HIV virion and is necessary to exert their anti-HIV activity^18,23,25,36–38^. Additionally, ssDNA binding is critical for deaminase activity. RNase A treatment is required to activate or enhance the deamination on ssDNA for A3H and several APOBEC members^24,29,32,33,39–41^, which suggests overlapping binding sites for both RNA and ssDNA substrates. The data reported so far suggest that APOBEC proteins can utilize diverse modes of binding to nucleic acids, all of which fulfill a number of different functions and regulations.

A3H, together with A3A, shows about three magnitudes stronger cytosine (C) and methylcytosine (mC) deaminase activity compared to other APOBEC members in *in vitro* activity assay using purified recombinant proteins^33,39,40,42,43^. Moreover, the selectivity for mC deamination of A3H is several times higher than that of A3A and other APOBECs^29,39,40^. While a detailed mechanism for the high activity and mC selectivity for A3H is not yet well understood, loop 1 and loop 7 have been shown to play major roles in regulating activity and mC selectivity in A3B and A3A^39,40^. Furthermore, the significance of mC deamination by APOBECs has not yet been fully characterized with respect to cellular function; however, the mC deamination activity associated with AID has been proposed as an alternative demethylation pathway for regulating methylation patterns in genomic DNA of mouse germ cells^44^, and for cell reprogramming in induced pluripotent stem cells^45–47^. Inadvertent deamination of genomic DNA by A3H and some other APOBECs has been associated with mutations in various types of cancer^35,48–52^.

In order to further our understanding of the structural basis of the biochemical functions of A3H, we performed structural and extensive biochemical studies on human A3H. We have obtained a 2.49Å crystal structure of an RNA-free monomeric A3H, which shows a uniquely long C-terminal helix 6 (h6) and a disrupted beta strand in the canonical five-stranded β-sheet core. Mutation of one loop 7 residue, W115, is critical for disrupting the RNA-mediated dimerization of A3H, yielding an RNA-free monomeric protein that still shows binding to nucleic acids and deaminase activity. By analyzing mammalian cell lysates expressing A3H, we show that the formation of HMW complexes of A3H in mammalian cells and the inhibition of deaminase activity depend on RNA binding. A3H has a highly positively charged surface covering the entire surface area where the Zn-active center and substrate binding loops are located. We show that multiple positively charged residues within this charged surface play an important role for RNA-mediated HMW formation and for RNA-dependent inhibition of deaminase activity. Furthermore, these positively charged residues regulate the subcellular distribution of A3H between nucleus and cytosol. Finally, we have identified multiple residues of loop 1 and loop 7 that contribute to overall deaminase activity as well as mC selectivity.

## Results

### Monomeric and dimeric forms of human A3H

**Figure 1:**
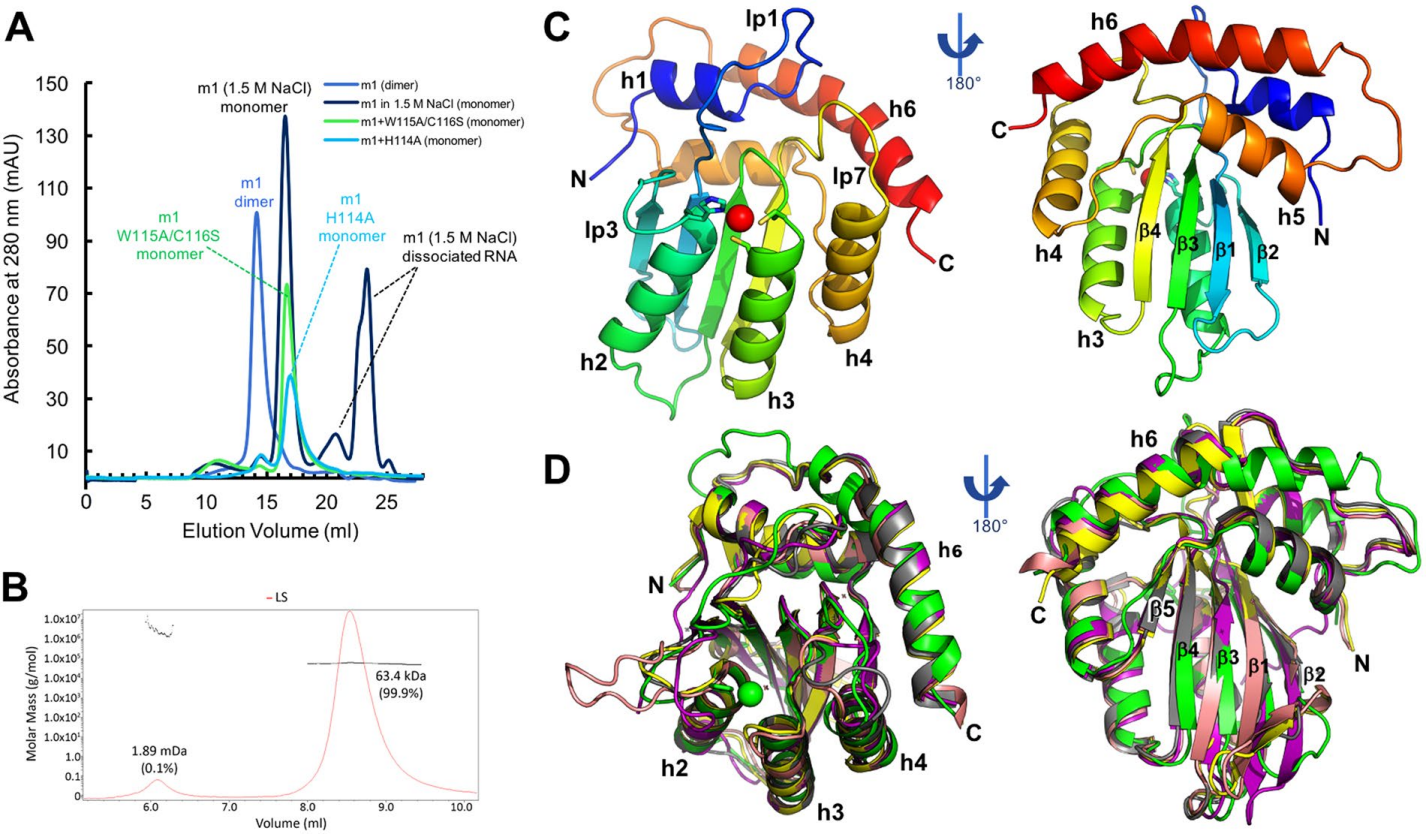
Protein purification and the overall structure of A3H. (**A**) SEC elution profiles of MBP-A3H dimeric and monomeric mutants on Superdex 200. A3H m1 forms a stable dimer after extensive RNase A treatment (blue). The purified m1 dimer can dissociate to monomer and free RNA after RNase A treatment followed by 1.5 M or higher salt buffer (black). The RNA-bound m1 dimer was disrupted by two sets of mutations on loop 7: H114A (m1+H114A) or W115A/C116S (m1+W115A/C116S), and clean monomers were purified from m1+H114A (light blue) m1+W115A/C116S (green). (**B**) MALS of MBP-fused m1+W115A/C116S mutant, showing the clean monomeric form. The expected molecular mass of a monomer is 63.2 kDa. (**C,D**) Crystal structure of A3H m1+W115A/C116S monomer mutant (**C**) and the superimposition of the A3H (green) with A3A (PDB: 4XXO, yellow), A3B-CD2 (PDB: 5CQI, salmon) and AID (PDB: 5W0R, purple) (**D**), with secondary structures indicated (Supplementary Figure S2A). The long helix 6 (h6), break of β5, and the long loop 1 of A3H can be visualized in panels C and D (Supplementary Figure S3A,B).

Human A3H tends to oligomerize not only inside cells, but also during purification^29,40^. Using the wild-type (WT) A3H haplotype II (referred to as A3H hereafter) with an MBP fusion tag at its N terminus, we were able to purify the WT A3H dimeric form that could be dissociated to monomer and free RNA through a 2 M high salt treatment and size exclusion chromatography (SEC) on a Superdex 200 column (Supplementary Figure S1). Due to the difficulty in generating the monomeric form of the WT A3H, extensive screening was conducted to search for A3H mutants that would produce a stable monodispersed A3H monomer for structural studies. Subsequently, three mutants were identified that could be purified to either dimeric or monomeric form: A3H m1, A3H m1 plus H114A (m1+H114A), and A3H m1 plus W115A/C116S (m1+W115A/C116S). While A3H m1 carries a set of 7 mutations (Supplementary Figure S2A) and produced a stable dimer form, stable monomeric forms can be isolated from both A3H m1+H114A and m1+W115A/C116S mutants (Fig. 1A,B). With these stable dimeric and monomeric forms of the A3H WT and mutants in hand, we performed crystallization trials either with the MBP tag or the cleaved forms, with or without nucleic acids, and obtained a high-quality crystal form of the cleaved A3H m1+W115A/C116S monomer that diffracted to 2.49 Å resolution (Table 1).

**Table 1:** Crystallographic data collection and refinement statistics.

	A3H
**Data collection**	
Space group	P2_1_
Cell dimensions	
*a*, *b*, *c* (Å)	46.749, 65.006, 65.540
*α*, β, γ (°)	90.00, 90.08, 90.00
Resolution (Å)	50–2.49 (2.58–2.49)*
*R*_sym_ or *R*_merge_	8.7 (49.1)
*I*/*σI*	19.6 (2.5)
Completeness (%)	98.8 (90.9)
Redundancy	6.2 (4.4)
Molecules per ASU	2
**Refinement**	
Resolution (Å)	50–2.49
No. reflections	13746
*R*_work_/*R*_free_	21.12/23.48
No. atoms	3009
Protein	2976
Ligand/ion	2
Water	31
*B*-factors	48.0
Protein	47.95
Ligand/ion	45.68
Water	52.72
R.m.s. deviations	
Bond lengths (Å)	0.002
Bond angles (°)	0.551

Structure was determined from a single crystal. *Highest-resolution shell is shown in parentheses.

### General structural features of human A3H monomer

The A3H m1+W115A/C116S monomeric structure was determined to 2.49Å resolution and refined to excellent statistics (Table 1). Each asymmetric unit (asu) contains two A3H molecules that have nearly identical structure for the core fold and the loops. This A3H structure, together with that of APOBEC2^53^, is perhaps the most divergent among known APOBEC structures so far^13,54–65^, which is consistent with the sequence analysis of the APOBEC family^66^. Notably, helix 6 (h6) of A3H extended six amino acid residues (1.7 turns) at its N-terminal side (Fig. 1C,D), making it the longest h6 among all APOBECs. The canonical short beta strand 5 (β5) of APOBEC proteins^53^ is not a typical strand in this A3H monomeric structure (Fig. 1D). The disruption of β5 appears to result from the proline residue (P132) that forms an outward-facing bulge and moves away from β4, disrupting the already short β5 (Supplementary Figures S3A, S3B), which may reflect an alternative conformation of β5 in A3H natural state. In addition to β5 strand, the A3H monomer contains a long loop 1 around the Zn-center (Fig. 1C, Supplementary Figure S2B). Other secondary structural features of the APOBECs are well preserved in A3H.

**Figure 2:**
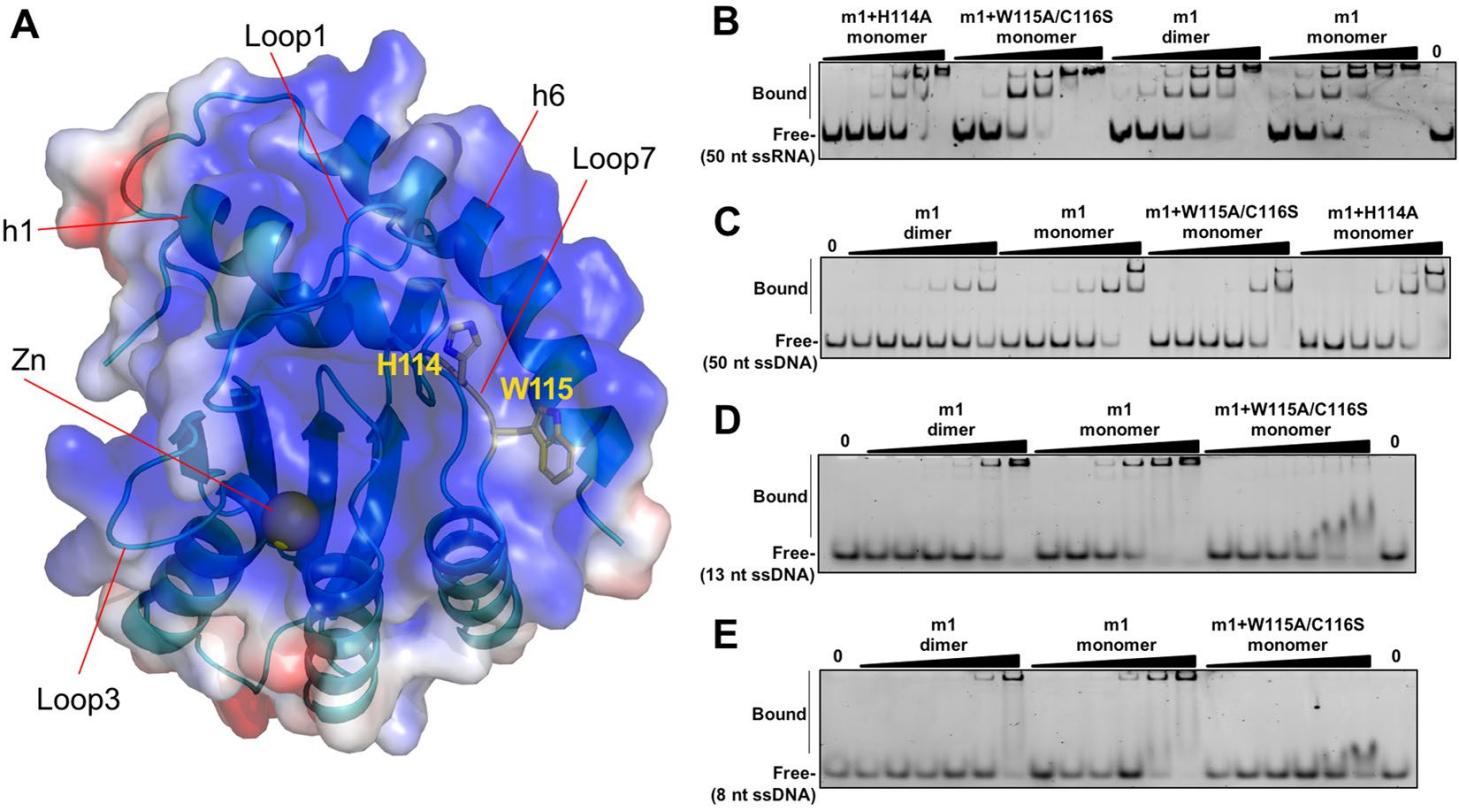
The positively charged surface and the nucleic acid binding property of A3H. (**A**) The positively charged surface around the Zn-active center of A3H, covering loop 1, 3, 5, 7, and helix 1 and 6 (h1, h6) and the Zn-center. The other end of the molecule is mostly neutral or negatively charged (Supplementary Figure S3C). The surface electrostatic potential colored according to calculated electrostatic potential of accessible surface area from −5 *kT/e* (red) to 5 *kT/e* (blue). The side chain of W115 was modeled based on the monomer structure. (**B–E**) Representative gel images of EMSA showing A3H mutants binding to 50 nt ssRNA (1 nM, **B**), 50 nt ssDNA (10 nM, **C**), 13 nt ssDNA (10 nM, **D**), and 8 nt ssDNA (10 nM, **E**). Quantification and estimate of binding affinity for each oligonucleotide are shown in Table 2.

The monomeric structure also shows that A3H is highly positively charged on one end around the Zn-active center (Fig. 2A), and more or less neutral and negatively charged on the other end (Supplementary Figure S3C). When compared with the active APOBEC domain structures using the same plotting scale, A3H shows the most extensive positively charged surface (Supplementary Figure S5). Other highly positively charged APOBEC proteins and domains include AID, A3A, and A3F-CD2.

During preparation of this manuscript, two structural studies of A3H dimer with RNA bound at the dimeric interface were published (PDB ID: 5W3V^63^ and PDB ID: 6B0B^67^). The overall structural features of our RNA-free monomeric A3H described here (PDB ID: 5W45) are very similar to those of the reported dimeric A3H structures. Even though our monomeric A3H structure contains nine point mutations, it superimposes well with 5W3V (pig-tailed macaque A3H, or pgtA3H) with an r.m.s.d of 0.598 for all atoms (Supplementary Figure S4), including h3 and h4 that are predicted to bind HIV Vif ^21^. The differences between the RNA-free hA3H and the RNA-bound pgtA3H structure are mostly reside in loops 1, 3, and 7. However, the superimposition with 6B0B (human RNA-bound A3H dimer) yielded a large r.m.s.d of 1.327, indicating significant differences (Supplementary Figure S4). It is worth noting that β5 is present in both dimeric A3H structures^63,67^ (Supplementary Figure S4). Therefore, it is possible that the disruption of β5 could also be the result of the 9 point mutations present in the A3H monomeric construct, even though the conformation around the mutated residues superimpose well with the pgtA3H structure (5W3V).

### Nucleic acid binding of A3H

The fact that high salt treatment of the dimer form yielded monomeric A3H and free RNA suggests that RNA binding can mediate dimer formation. In addition, stable monomeric A3H can be obtained simply by mutating H114 or W115/C116 (as mutants A3H m1+H114A and m1+W115A/C116S) (Fig. 1A,B), suggesting that H114 and W115 participate in nucleic acid binding and their interactions with RNA are critical for dimer formation. H114 and W115 on loop 7 of A3H are located around the center of a highly positively charged surface (Fig. 2A). To compare the binding affinity of the dimeric and monomeric forms of A3H to nucleic acids, we employed electrophoretic mobility shift assay (EMSA). It was expected that a difference in binding would reflect the difference of the mutated H114 and W115 and their contribution to nucleic acid binding.

We focused our binding study on various single stranded oligonucleotides as our initial investigation using both EMSA and SEC assays revealed no detectable binding to dsDNA or dsRNA by the dimeric and monomeric forms of A3H (data not shown). We tested the binding of a 6-FAM labeled 50 nucleotide (nt) ssRNA and ssDNA (Fig. 2B,C), containing a mixed sequence with no predicted secondary structure. Surprisingly, the results revealed relatively strong binding to both 50 nt RNA or DNA for all of the A3H constructs tested, with K_d_ values between 8–34 nM for RNA and 22–68 nM for DNA (Table 2), and the general trend of the binding affinity toward RNA or DNA was very similar. Only small differences in binding affinity for the 50 nt RNA or DNA were observed between the dimeric construct (m1) and the monomeric constructs (m1+H114A or +W115A/C116S) (Fig. 2B,C, Table 2). When comparing the affinity of different monomeric forms to the 50 nt RNA substrate, the m1 monomer form (converted from the dimer form by high salt) showed a K_d_ of ~8 nM, which is slightly tighter binding than the 12 nM K_d_ for the W115A/C116S mutant, and the 34 nM for the H114A mutant (Table 2). Nonetheless, both W115A/C116S and H114A mutations completely disrupted the RNA-mediated dimer formation to give a fully monomeric form (Fig. 1A). When comparing between the dimeric and monomeric form of the same m1 construct, the monomeric form showed stronger binding than the dimeric form for both 50 nt RNA and DNA (Table 2).

**Table 2:** ssDNA and ssRNA binding by A3H dimeric and monomeric mutants.

A3H constructs^1^ (oligomer state)	K_d_ (nM)			
	m1 (dimer form)	m1 (monomer form)	m1+W115A/C116S (monomer only)	m1+H114A (monomer only)
ssRNA (FAM-50 nt)	14.3 ± 1.0	8.4 ± 0.4	12.6 ± 1.7	34.9 ± 1.8
ssDNA (FAM-50 nt)	68.5 ± 3.3	35.0 ± 1.0	39.8 ± 2.2	22.2 ± 1.3
ssDNA (FAM-13 nt)	1,357 ± 37	272 ± 8	497 ± 11	
ssDNA (FAM-8 nt)	2,846 ± 134^2^	983 ± 23^2^	2,960 ± 571^2^	
	**Hill coefficient (cooperativity)** ^**4**^			
ssRNA (FAM-50 nt)	1.9 ± 0.2	1.9 ± 0.2	2.8 ± 0.9	2.4 ± 0.2
ssDNA (FAM-50 nt)	2.6 ± 0.3	4.6 ± 0.7	3.8 ± 0.6	2.2 ± 0.2
ssDNA (FAM-13 nt)	3.7 ± 0.3	4.3 ± 0.6	3.5 ± 0.2	
ssDNA (FAM-8 nt)	2.7 ± 0.2	3.7 ± 0.3	—^3^	

The K_d_ values were obtained based on EMSA results, which should only be considered as approximate estimates of the binding affinity, especially for the shorter 13 nt and 8 nt oligomers. ^1^A3H mutants m1 and m1+W115A/C116S contain the catalytic residue E to A mutation. However, when the catalytic E is not mutated, both A3H m1 and m1+W115A/C116S are highly active, with m1+W115A/C116S being about 2.7-fold less, and m1 being about 3.5-fold more active than WT A3H (see Supplementary Table S1). ^2^The EMSA gel shift bands are smears or less defined bands for the 8 nt oligomer, and the quantification of binding is an approximate estimate. ^3^No obvious cooperativity was observed. ^4^All constructs of A3H showed some level of cooperativity in nucleic acid binding, but it is difficult to define the degree of cooperativity based on the estimated Hill coefficient.

We then tested the ssDNA binding with two short oligonucleotides, 13 nt and 8 nt, to compare the dimeric/monomeric m1 and monomeric m1+W115A/C116S (Fig. 2D,E). If compared between the two monomeric forms, the m1+W115A/C116S monomer mutant showed much weaker binding and different shift pattern as the oligonucleotide gets shorter. With the FAM-13nt ssDNA, the binding affinity of W115A/C116S mutant had a K_d_ of 497 nM, whereas m1 was 272 nM (Table 2). With the FAM-8 nt ssDNA, however, the K_d_ of W115A/C116S monomer mutant was 2.96 µM; about a 3-fold drop in binding affinity compared to 983 nM for m1 monomeric construct. Again, if compared between the dimeric form and monomeric form of the same m1 dimer construct, the monomeric form showed stronger binding than the dimeric form for shorter oligomers (Table 2). Because of the similarity of binding between 50 nt ssRNA and ssDNA, the phenomenon observed for the shorter ssDNA is likely to be similar for the shorter ssRNA as well. Additionally, all A3H constructs showed some level of cooperativity in their binding to the ssDNA/RNA of different lengths, but it is difficult to define the degree of cooperativity based on the estimated Hill coefficient.

### Formation of A3H HMW species in HEK293T cells

**Figure 3:**
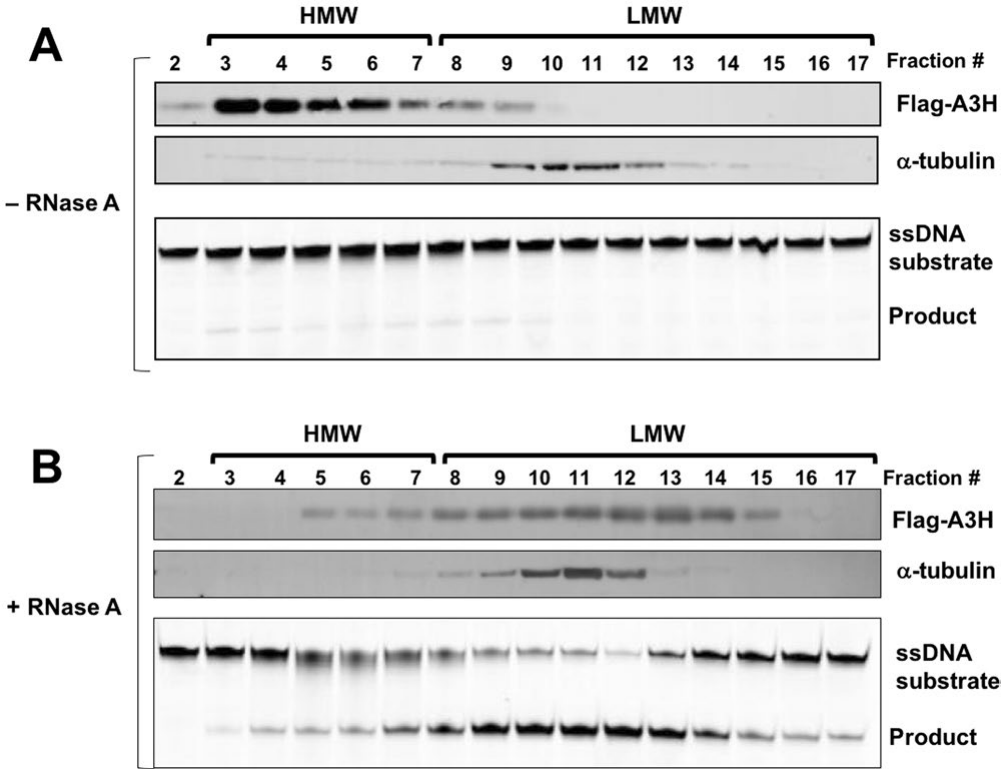
Multimerization of A3H in HEK293T cells and RNA-dependent inhibition of A3H deaminase activity. (**A**) A3H formed enzymatically inactive high molecular weight (HMW) ribonucleoprotein complex. Cell lysates of HEK293T cells expressing A3H, untreated or treated with RNase A, were fractionated by SEC on Superdex 200 column and then analyzed by Western blot and deaminase activity assay. HMW complexes were observed, and essentially no obvious deaminase activity was detected. (**B**) After RNase A treatment, the HMW complexes of A3H were converted to enzymatically active low molecular weight (LMW) species. α-tubulin is an endogenous control.

Non-substrate nucleic acid binding by APOBEC proteins, especially through binding of RNA that is freely available inside cells, may be a general factor for the multimerization of these enzymes and RNA-mediated inhibition of deaminase activity^33,36,37^. Here we examined the multimerization status of an N-terminal FLAG-tagged A3H (FLAG-A3H) expressed in mammalian HEK293T cells using a cell fractionation assay, and tested the effect of RNase A treatment of the cell lysates on both the oligomeric status and deaminase activity. The cell lysates of 293T cells overexpressing FLAG-A3H either before or after RNase A treatment were fractionated by SEC on an Superdex 200 column, and fractions across the elution range were analyzed by SDS-PAGE and Western blot with an anti-FLAG monoclonal antibody (mAb) to detect the multimerization status of A3H (Fig. 3A,B). At the same time, each fraction was also tested for deaminase activity. The results showed that, without RNase A treatment, A3H in the cell lysates eluted mostly in high molecular weight (HMW) fractions, and very little deaminase activity was detected across these fractions, being barely above background levels (Fig. 3A). However, in the RNase A-treated cell lysates, the HMW species dissociated to the LMW species, and deaminase activity was detected across all fractions, with high activity associated with the LMW fractions. These results indicate that RNA binding of A3H is involved in multimerization and inhibition of deaminase activity of A3H *in vivo*. These results are consistent with the strong binding of A3H to nucleic acids and the RNA-mediated dimerization observed previously.

### Role of positively charged residues in subcellular localization of A3H

**Figure 4:**
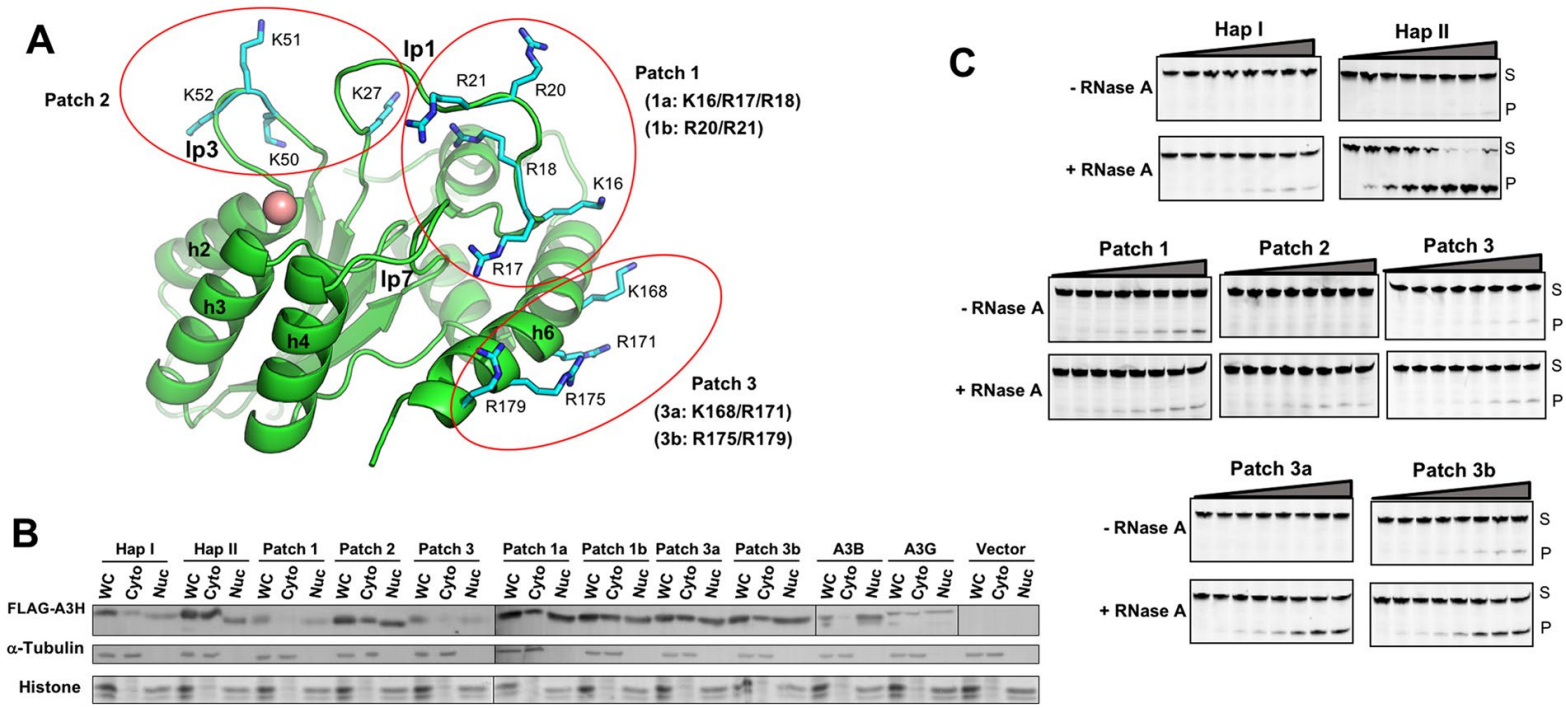
Positively charged patches are important for subcellular localization and deaminase activity of A3H. (**A**) A3H structure showing the positively charged residues mutated in three patch mutants (patch 1–3). (**B**) Cell fractionation analysis of A3H and various mutants, showing the distribution between nucleus and cytosol in HEK293T cells. Transfected 293T cells expressing wild-type A3H hap I, hap II, and various hap II mutants were fractionated into whole cell (WC), cytoplasmic (Cyto) and nuclear (Nuc) fractions. A3B (mostly nucleus) and A3G (both cytoplasm and nucleus) were also used as controls. FLAG-A3H proteins in each fraction were analyzed by Western blot. (**C**) The deaminase assay of selected A3H mutants using the cell lysates of transfected HEK293T cells with or without RNase A treatment. The deaminase reaction was performed with cell lysate range of 0–6 μg (total protein amount, 2-fold dilutions from 6 μg) and 300 nM ssDNA.

On the highly positively charged surface of A3H, there are a total of thirteen arginine and lysine residues (Fig. 4A), which can be grouped into three patches (patch 1: K16A/R17A/R18A/R20A/R21A, patch 2: K27A/K50A/K51A/K52A, and patch 3: K168A/R171A/R175A/R179A) based on their location. In order to test the contribution of these positively charged residues to RNA-mediated HMW formation in cells, we generated the corresponding patch mutants by replacing these three groups of residues to alanine (Fig. 4A), and then tested multimerization status in HEK293T cell lysates. When we tried the cell lysate analysis of the HMW formation for these patch mutants (patch 1–3), however, there were too little patch mutant proteins in the soluble fractions of the cell lysates for the multimerization analysis through SEC fractionation. Upon analysis of the total proteins expressed, however, the total proteins of the A3H patch mutants in the whole cell lysates were comparable to that of the WT (Supplementary Figure S6). To test if the reduced patch mutant proteins in the soluble fraction could be due to relocalization from cytosol to nucleus, we applied subcellular fractionation accompanied with Western blot to examine the distribution of the WT A3H hap I, hap II, and the patch mutants. Previously it has been shown that A3H hap I predominantly localizes in nucleus, whereas A3H hap II mainly in cytosol^22,23^. In addition, we included two controls, FLAG-A3B known to be localized in the nucleus and FLAG-A3G localized in the cytosol^68^.

The subcellular fractionation analysis showed that A3H hap I was mostly localized in the nucleus (~71%), consistent with previous reports^22,23^. Comparing to hap I, A3H hap II had a reduced level in nucleus (~50%) (Fig. 4B, Table 3). Interestingly, two of the three patch mutants, patch 1 and 3, showed significantly higher distribution in the nucleus than WT A3H, with ~80% for both (Table 3). Patch 2 mutant showed less profound change, but still increased nuclear localization to ~65% (Fig. 4B, Table 3). These results suggest that the positively charged residues in patch 1 and patch 3 play a major role in mediating subcellular localization.

**Table 3:** Subcellular distribution of A3H positively charged patch mutants.

A3H protein		Nuclear localization Nuclear/(Nuclear + Cytosol)
Hap I		0.71 ± 0.05
Hap II		0.50 ± 0.14
Hap II m1+W115A		0.77 ± 0.03
A3H hap II mutants	Patch 1 (K16/R17/R18/R20/R21 to A)	0.80 ± 0.03
	Patch 2 (K27/K50/K51/K52 to A)	0.65 ± 0.07
	Patch 3 (K168/R171/R175/R179 to A)	0.80 ± 0.10
	Patch 1a (K16/R17/R18 to A)	0.68 ± 0.07
	Patch 1b (R20/R21 to A)	0.51 ± 0.02
	Patch 3a (K168/R171 to A)	0.53 ± 0.003
	Patch 3b (R175/R179 to A)	0.68 ± 0.11

The subcellular distribution of different mutants and WT A3Hs was estimated based on the Western blot results of cell fractionation. The cell fractionation results showed that the distribution of the α-tubulin was detected in the cytosol and the histones in the nuclear fraction on the Western blots (Fig. 4B, and Supplementary Figure S6), indicating a clean separation of cytosol and nuclear fractions. S.D. was estimated from data collected in two to three independent experiments.

To further examine if any specific residues within patch 1 and patch 3 are important in regulating subcellular localization, we made four additional mutants, two within patch 1 (patch 1a: K16A/R17A/R18A and 1b; R20A/R21A) and two within patch 3 (patch 3a: K168A/R171A and 3b: R175A/R179A) (Fig. 4A, Table 3). The results showed that, even though patch 1 mutant has a major change in its nuclear localization to 80%, patch 1a was lowered to 68% and patch 1b was similar to the WT (51%, Table 3). Interestingly, similar results were also seen with patch 3a and 3b. While patch 3a showed similar subcellular localization as the WT (53%), patch 3b showed predominant nuclear localization (~68%), even though still less than the full patch 3 mutant (Fig. 4B, Table 3). In addition, A3H carrying W115A mutation (m1+W115A) also showed predominant nuclear localization (Supplementary Figure S6). The results for R175 and W115 affecting the subcellular localization is consistent with the newly published dimeric A3H structure report^67^. These results indicate that W115, K16A/R17A/R18A, and R175/R179 play an important role in determining subcellular localization, and the rest of the positively charged residues, even though had no major effect by themselves, can work together additively to affect subcellular localization.

We tested if there is any difference in RNA-mediated inhibition of deaminase activity for the patch mutants, as an alternative way of assessing RNA binding. In addition to the three patch mutants, two of the sub-patch 3 mutants were also included in this analysis: patch 3a (K168A/R171A), which had shown no obvious change in subcellular distribution, and patch 3b (R175A/R179A) that had shown increased nuclear localization (Table 3). The results showed that, while WT A3H hap II displayed high activity only after RNase A treatment, hap I, and three patch mutants (patch 1–3), all had significantly reduced deaminase activity with or without RNase A treatment (Fig. 4C), suggesting that these three patch mutants may have impaired binding to substrate ssDNA for deamination. Of note, patch 1 mutant showed comparable levels of activity regardless of RNase A treatment, even though overall activity decreased compared to WT, suggesting that inhibition of activity by RNA is attenuated in this mutant. For patch 3a (K168A/R171A), which had a similar subcellular distribution as WT, no activity was observed without RNase A treatment, and deaminase activity was only detected after the treatment, suggesting that inhibitory RNA binding is still present in this mutant. Conversely, patch 3b (R175A/R179A), which had shown increased nuclear localization, showed detectable level of activity without RNase A treatment, and RNase treatment did not show a significant increase of activity. Taken together, the activity assays with or without RNase A treatment of the positively charged patch mutants suggest that disruption of nucleic acid binding (either ssDNA or RNA) plays a role in subcellular distribution.

### Importance of loop 1 for deaminase activity and mC selectivity of A3H

**Figure 5:**
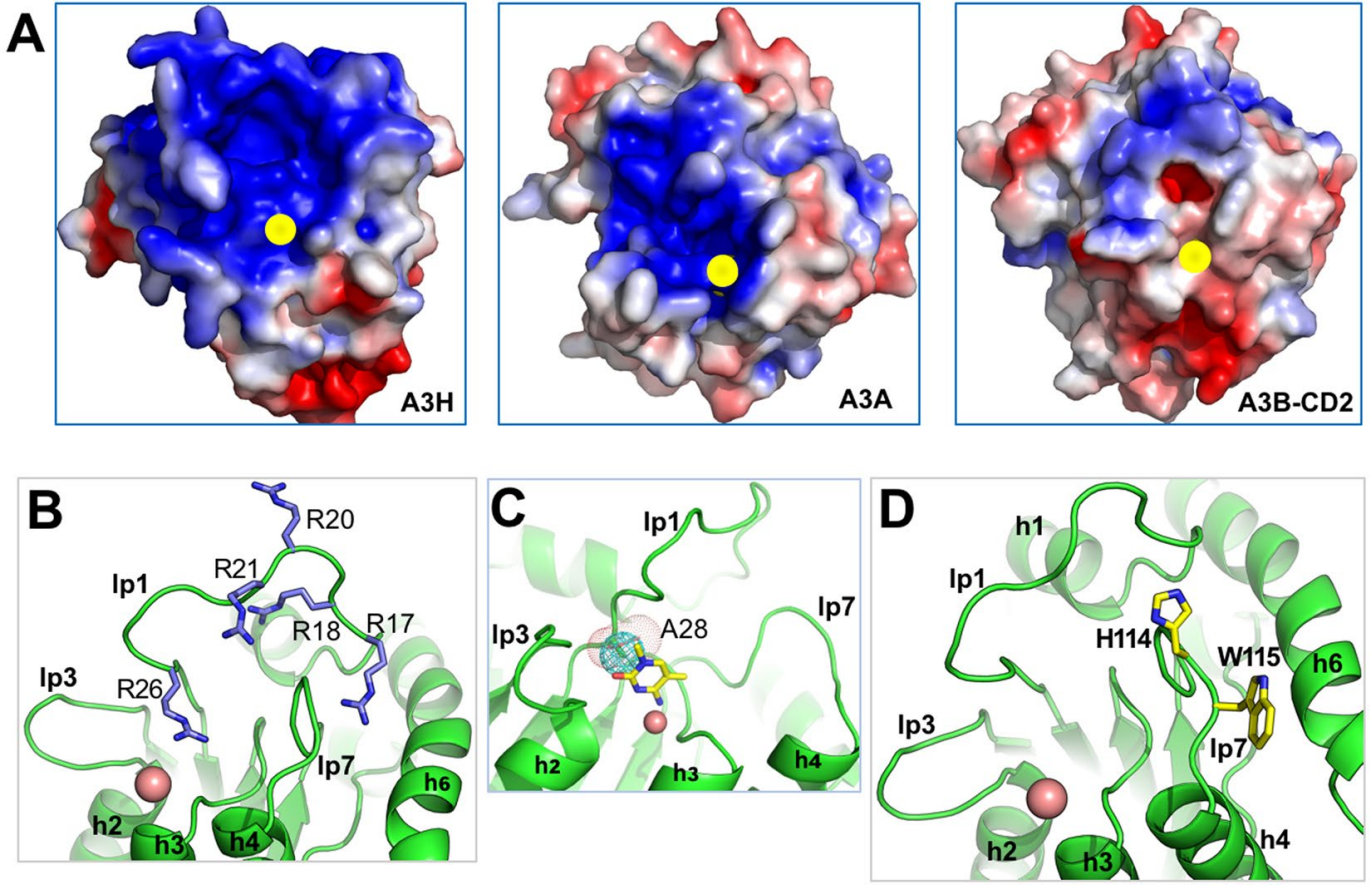
Comparison of the charged surface and the residues on loop 1 and loop 7 surrounding the Zn-active site center of A3H, A3A (PDB: 4XXO), and A3B-CD2 (PDB: 5CQI). (**A**) The charge distribution around the Zn-active site, showing that the Zn-center of A3H and A3A, but not of A3B-CD2, is surrounded by positively charged surface. The surface electrostatic potential colored according to calculated electrostatic potential of accessible surface area from −5 *kT/e* (red) to 5 *kT/e* (blue). (**B**) Five arginine residues on loop 1 of A3H that affect deaminase activity and mC selectivity. (**C**) Position of A28 (blue dotted sphere) at the Zn-active center pocket, located on the side of the modeled target C in the pocket. The space that would be occupied by a larger T/S at the same position in other APOBECs is represented by red dotted sphere that may pack tighter with the target C. (**D**) The residues on loop 7, H114 and W115 are shown in sticks. The side chain of W115 was modeled based on the monomer structure.

It has been shown previously that A3H and A3A have about three orders of magnitude stronger deaminase activity than other APOBECs by an *in vitro* assay using purified recombinant proteins^29,39,40,42,69^. Comparing the surface charge distribution around the Zn- center of all catalytically active APOBEC domains, A3H and A3A are the only two APOBECs having their Zn-center surrounded by a predominantly positively charged surface area (Fig. 5A, Supplementary Figure S5). The highly positively charged environment around the Zn-center in A3H and A3A may help attract any ssDNA substrates directly to the Zn-center for efficient deamination. As a comparison, other active APOBEC domains have positively charged surfaces located some distance away from the Zn-center (Fig. 5A, Supplementary Figure S5), suggesting that substrate DNA may initially bind to the positively charged area away from the Zn-center and then extend the target C to the active site for deamination, as in the case reported for A3F-CD2 in complex with ssDNA^70^. Thus, there may be a relationship between the charge distribution around the Zn-center and deaminase activity levels observed for different members of the APOBEC family.

**Figure 6:**
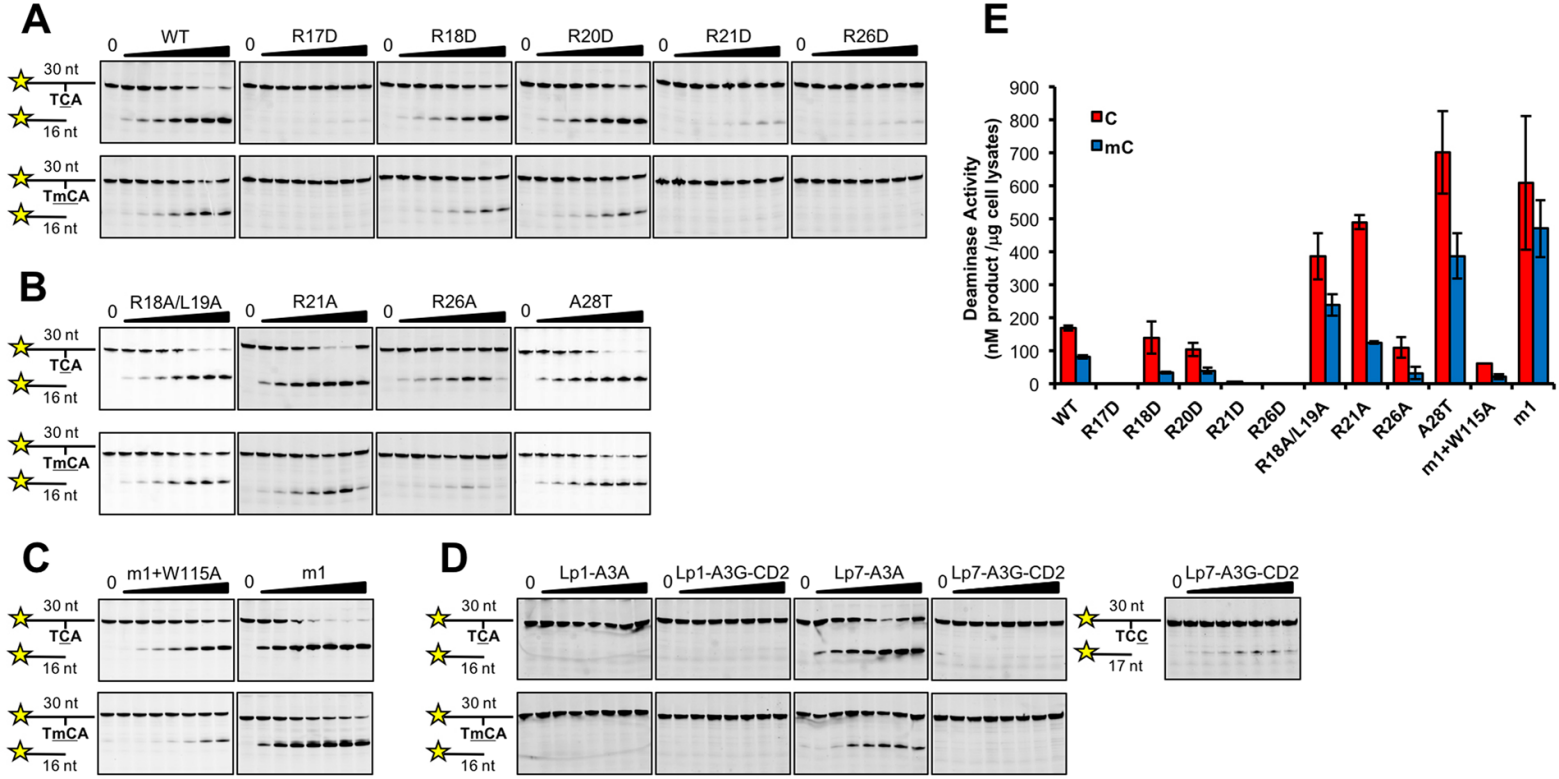
The deaminase activity assay on C and mC of A3H and various mutants on loop 1 and loop 7. (**A–D**) Representative gels of the deaminase assay of the A3H loop 1 and loop 7 mutants, including point mutations on loop 1 (panels A,B), on loop 7 (panels C), and loop swapped mutants of loop 1 and loop 7 (panel D), using cell lysates of transfected HEK293T cells. The deaminase reaction was performed with cell lysate range of 0–6 μg (total protein amount, 2-fold dilutions from 6 μg) and 300 nM ssDNA. (**E**) A bar graph representation of the quantification of the deaminase assay results from (**A–C**) (also see Supplementary Table S1).

Both A3H and A3A have been shown to similarly have strong mC deaminase activity. However, the mC selectivity factor (defined as mC/C specific activity x 100) of A3H is around 50, the highest among all APOBECs^29,40^. Here with the structure of A3H at hand, we tried to understand structural elements important for the high deaminase activity and high mC selectivity observed for A3H. Previous studies showed that loop 1 is important in determining the deaminase activity and mC selectivity for engineered A3B-CD2 and A3A^39,40^. In this study, we have examined the role of A3H loop 1 with regards to deaminase activity and mC selectivity using cell lysates from mammalian cells expressing A3H. A3H loop 1 is highly charged, with five arginine residues (Fig. 5B) located in the previously mutated patch 1 area (Fig. 4A). We first generated R to D point mutations for each of these five residues, and the results showed that mutants R17D, R21D, and R26D essentially lost all deaminase activity (Fig. 6A,E), demonstrating that negatively charged residues at these positions of loop 1 abolished deamination. The other two mutants, R18D and R20D, decreased the activity by ~20–40% on C deamination, and by ~40–50% on mC deamination (Fig. 6A,E, Supplementary Table S1). The mC selectivity factor of R18D decreased significantly to 24.5 (from 48.3 for WT A3H) (Supplementary Table S1), whereas that of R20D only had a moderate decrease.

We further tested the effect of alanine substitution on a few selected positions on loop 1, including R21A, R26A and R18A/L19A. In contrast to R21D and R26D mutants that showed complete loss of activity, R21A had even higher deaminase activity than WT, and R26A showed activity comparable to WT (Fig. 6B,E, Supplementary Table S1). Interestingly, despite the high C deaminase activity for R21A and R26A, both had a significant decrease of mC selectivity, to 25.7 and 30.5, respectively (Supplementary Table S1). Surprisingly, R18A/L19A showed about 2-fold higher C deaminase activity than WT A3H, and its mC selectivity factor also increased to 62 (Fig. 6B,E, Supplementary Table S1). These data suggest that not only the positions, but also the residue types on loop 1 can have a significant effect on deaminase activity as well as mC selectivity. Moreover, the increase of C deaminase activity of a particular mutant can result in both a highly reduced mC selectivity (as in R21A) or significantly increased mC selectivity (as in R18A/L19A).

A sequence alignment of the APOBEC proteins also revealed a unique residue, A28, on the C-terminal end of A3H loop 1, that is either T or S on all other active APOBEC domains (Supplementary Figure S2B). In the 3D structure, A28 occupies the same position as T that packs on the back side of the target cytosine base at the Zn-center pocket (Fig. 5C), and presumably could help to stabilize the C base inside the pocket for deamination. In order to test whether this unique A28 of A3H would provide more room for the larger mC inside the pocket and allow for higher mC selectivity, we made an A28T mutant of A3H, and the results of the subsequent activity assay showed that A28T had higher deaminase activity on both C and mC than WT A3H (Fig. 6B,E), presumably holding the base tighter for more efficient deamination. However, the A28T mutant showed no significant change in mC selectivity (Supplementary Table S1), indicating that A28 is not one of the factors accounting for the higher mC selectivity in A3H.

### The W115 on loop 7 is important for deaminase activity and mC selectivity of A3H

Because our data presented earlier suggests that W115 of loop 7 has a role in the RNA-mediated dimerization of A3H (Fig. 1A), a result that is also confirmed by the recent reports^63,67^, we examined the role of W115 in deamination and mC selectivity using the A3H m1 construct (containing WT residue W115) and m1+W115A, overexpressed in 293T cells. The results showed that, compared to WT A3H, the deaminase activity of m1 on C and mC increased by about 3.5-fold on C and 5.7-fold on mC, resulting in an increased mC selectivity factor to 77. However, the deaminase activity of m1+W115A decreased by about 2.7-fold on C and 3.9-fold on mC compared to WT A3H, resulting in a lower mC selectivity factor (Fig. 6C,E, Supplementary Table S1). These results indicate that W115 on loop 7 plays an important role not only in RNA-mediated dimerization of A3H, but also in interacting with substrate ssDNA for deaminase activity and mC selectivity.

### Effects of loop swapping of loop 1 and loop 7

Because the mutational studies showed that residues on loop 1 and loop 7 of A3H play important roles in determining the deaminase activity and mC selectivity, we also tested functions of loop 1 and loop 7 of A3H by swapping them with the equivalent loops from A3A, a highly active APOBEC, or A3G-CD2, an APOBEC with low activity. The results showed that swapping loop 1 of A3H with either A3A or A3G-CD2 resulted in a complete loss of activity (Fig. 6D,E, Supplementary Table S1), indicating that loop 1 from A3A and A3G-CD2 are not compatible with the active center configuration of A3H for deaminase activity, likely disrupting the correct substrate ssDNA binding mode necessary for deamination.

However, swapping loop 7 of A3H with A3A showed deaminase activity comparable to that of WT A3H, except that the mC selectivity factor is reduced to 29 (Fig. 6D,E, Supplementary Table S1). Loop 7 swapping with that of A3G-CD2, in contrary, resulted in a loss of activity on TCA motif. Since loop 7 of A3G-CD2 is shown to favor the TCC motif for deamination^71,72^, the deaminase activity using substrate ssDNA containing TCC motif did show low activity that is barely above backgrounds (Fig. 6D). The loop 7 swapping results indicate that loop 7 of A3H can be replaced with that of A3A, but not of A3G-CD2. Taken together, it suggests that loop 1 and loop 7 of A3H are both important for the robust deaminase activity and mC selectivity, but loop 1 is more sensitive to changes than loop 7, which is consistent with the results shown earlier, where several point mutations on loop 1 abolished A3H activity.

## Discussion

Here we report the crystal structure of a human A3H monomer mutant and the structure-guided biochemical studies. The overall structure of this A3H monomeric form is conserved with those of other known structures of APOBEC proteins, and many of its detailed features are consistent with those of the two recently reported dimeric RNA-bound A3H structures (pgtA3H^63^ and hA3H^67^). The A3H structure has some unique features in that it has an extended helix 6 (h6) and a shortened strand 5 (β5) (Fig. 1, Supplementary Figure S3). In addition, A3H has the longest loop 1 and shortest loop 3 around the Zn-active center. Furthermore, A3H has the most extensive positively charged surface around the Zn-active center among all catalytically active APOBEC domains determined so far (Supplementary Figure S5).

In order to obtain A3H crystals, WT and mutant A3H proteins were purified as dimeric form with RNA bound or monomeric form from *E. coli*. (Fig. 1A, Supplementary S1). Using high salt treatment, we could convert the dimeric A3H into a stable monomeric form by dissociating the bound RNA (Fig. 1A, Supplementary S1). Stable monomeric A3H was also obtained by making H114A or W115A/C116S mutations (Fig. 1A,B), which presumably broke the dimer interaction by disrupting binding to RNA. While preparing this manuscript, two dimeric structures of A3H were published^63,67^, both of which show that RNA binding is important for dimer formation. Our data showing the conversion of dimeric A3H into monomeric form by dissociating RNA in high salt condition or by H114A or W115A/C116S mutations are consistent with the reported RNA-mediated A3H dimer structures^63,67^.

RNase A treatment is known to be required for activating A3H activity. Here we show that RNase A treatment of mammalian cell lysates expressing A3H not only activated A3H deaminase activity, but also converted the HMW form of A3H present in the soluble cell lysates into LMW species based on SEC fractionation (Fig. 3A,B). These results suggest that RNA binding by A3H plays a role in multimerization and HMW ribonucleoprotein complex formation in mammalian cells. This is similar to the case of A3G^73^, but is different from the case of A3B, where the HMW complex of A3B is insensitive to RNase A treatment, even though RNase A treatment can greatly enhance its deaminase activity^55^. This RNA dependent inhibition of deaminase activity and the need of RNase A treatment to obtain relatively RNA-free and more enzymatically active protein was first observed for Activation-induced deaminase (AID)^33^, and later found to be a common feature for other active APOBEC members.

The positively charged surface of A3H has thirteen R/K residues that can be grouped into three patches (Fig. 4A). Interestingly, mutations of these three patches, patch 1, 2, and 3, changed subcellular distribution of A3H, with patch 2 showing modest increase of nuclear localization to 65%, and patch 1 and patch 3 showing increase to 80% nuclear localization. These patch mutants carrying mutations of the positively charged residues to alanine had greatly reduced deaminase activity (Fig. 4C), indicating a loss of substrate binding. In addition, these mutants did not show a significant difference in their deaminase activity with or without RNase A treatment, which suggests that RNA binding may not be a factor impacting the deaminase activity. Interestingly, when the positively charged residues in groups of two or three residues within patch 1 (patch 1a, 1b mutants) and patch 3 (patch 3a, 3b mutants) are mutated, only patch 1a (K16/R17/R18 to A on loop 1) and 3b (R175A/R179A on helix 6, Fig. 4A, Table 3) significantly changed the distribution to the nucleus, while the other two (1b and 3a) had no obvious change of subcellular distribution. K16/R17/R18 in patch 1a and R175/R179 in patch 3b are involved in binding to RNA in the reported RNA-bound A3H structures^63,67^. However, the positively charged residues mutated in patch 1b (R20/R21) and patch 3a (K168/R171) are also involved in binding to the RNA but has no effect on change of subcellular localization. Furthermore, the four positively charged residues in patch 2 mutant has no major role in binding to the RNA in the A3H dimer structures^63,67^, yet patch 2 mutant showed obvious change of subcellular distribution. These results suggest that while RNA binding may play a role in affecting subcellular localization, there may be other factors affecting the nuclear localization. Additionally, the results also demonstrated that not all positively charged residues are equal in affecting subcellular localization. K16/R17/R18, R175 and R179 have more important role than other positively charged residues in subcellular localization, and the rest of the positively charged residues can act additively to impact subcellular localization.

The extensively positively charged surface areas around the Zn-center of A3H and A3A suggests that nucleic acids should be able to bind directly to the active site pocket, which may explain why A3H and A3A are the two APOBECs with higher activity than other members of the APOBEC family. Interestingly, the monomeric and dimeric mutants of A3H showed comparable K_d_ values for both 50 nt ssRNA and ssDNA. However, the monomeric mutant (m1+W115A/C116S) showed much less binding to 13 nt and 8 nt ssDNA than the dimeric mutant (m1) that can be converted to monomeric form through a high salt treatment (Table 2). The observation that the monomeric mutant containing W115A mutation showed much less binding to shorter nucleic acids may be in part because the hydrophobic interactions between the nucleotide base and the W115 side chain have a significant contribution to the binding of shorter nucleic acids, in addition to the charge-charge interactions through the multiple R/K residues. When the nucleic acids are sufficiently long, they can then bind to multiple sites across the positively charged surface, reducing the contribution of W115 to the overall binding affinity.

A3H has been shown to be highly catalytically active in C deamination, but also has the highest mC selectivity. Among the active center loops (i.e. loops 1, 3, 5 and 7) of APOBECs, loop 5 is highly conserved, while loops 1, 3 and 7 are variable among APOBEC members. The loop 3 of A3H has only four residues, making it the shortest among all active APOBECs. It is likely that such a short loop adopts a consistently open configuration, as seen in the non-substrate binding structure. However, loop 1 of A3H is the longest and most divergent in sequence among all active APOBECs, which makes it unique to A3H. By comparison, the other highly active APOBEC, A3A, has the shortest loop 1. There are also some unique sequence features on loop 1 and loop 7 of A3H: there are a total of seven R/K residues on loop 1 and a unique hydrophobic W (W115) on loop 7 (Supplementary Figure S2). Point mutations on five of the loop 1 R/K and the loop 7 W115 revealed that they all play a role in deaminase activity and mC selectivity, even though their relative contributions vary to some extent. Loop 1 and loop 7 have very different conformations in the apo-A3H structure reported here and the newly reported RNA-bound A3H structures, suggesting both loops are flexible and can adopt different conformations for binding RNA or ssDNA substrates, which may enable A3H to bind and orient the mC at the active site pocket for efficient deamination. While the detailed mechanisms likely require further co-crystal structures of A3H with various ssDNA substrates, the results shown here suggest that multiple residues on loop 1 and loop 7 contribute to the high activity and high mC selectivity in deamination, which is consistent with previous report on A3B-CD2 and A3A.

In summary, we describe a high-resolution structure of human A3H monomer and extensive structure-guided mutational analysis in order to understand the structural basis of its biochemical functions. This A3H structure, together with that of APOBEC2, is perhaps the most divergent from other APOBEC structures determined so far, with unique structural features including a longer C-terminal helix 6 (h6), a disrupted β5 strand of the canonical five-stranded β-sheet core, and a long loop 1 around the Zn-center. Mutation of a single loop 7 W115 residue disrupted the RNA-mediated dimerization of A3H to produce a clean monomeric form that still possessed nucleic acid binding and deaminase activity. A3H expressed in mammalian cells showed an RNA-dependent HMW complex formation and RNase A dependent deaminase activity. A3H has a highly positively charged surface containing multiple positively charged residues that play a critical role in the subcellular localization of A3H between the nucleus and cytosol. Multiple residues on loop 1 and loop 7 of A3H play a role in the overall deaminase activity and the mC selectivity. These structural and functional studies of A3H contribute to our further understanding of the biochemical functions in nucleic acid binding, multimerization, subcellular localization and mC selectivity of this important APOBEC deaminase enzyme.

## Methods

### Construction of plasmids

The coding sequences for the full-length human APOBEC3H hap II (GenBank accession: ACK77776) were codon-optimized for the expression hosts (*Escherichia coli* and human HEK293T) and synthesized by Invitrogen GeneArt Gene Synthesis (Thermo Fisher). The coding sequence of A3H hap I (GenBank accession: NP_001159474) was derived from that of A3H hap II through site-directed mutagenesis. A3H hap II constructs for crystallization trials and *in vitro* biochemistry study were generated in pMAL-c5X vector (NEB) with the N-terminal MBP tag and with or without PreScission Site. A3H hap I and hap II constructs for human cell-based study were generated in pcDNA 3.1(+) mammalian expression vector (Thermo Fisher) with an N-terminal FLAG tag. Cloning and mutagenesis were conducted with In-Fusion HD Cloning Plus and CloneAmp HiFi PCR Premix (Takara).

### Protein expression and purification

The expression protocol of various MBP-fused A3H constructs in *E. coli* was similar to the previously published protocol^55^. Briefly, *E. coli* cells harboring the A3H expression vectors were grown at 37 °C to about OD_600_ 0.2–0.3 and further growth was continued at 14–16 °C. IPTG at 0.1 mM was added when OD_600_ reached about 0.6–0.8 for overnight induction. To purify dimer and monomer of MBP-fused wild-type A3H hap II, *E. coli* cells expressing MBP-fused A3H hap II were harvested and lysed in buffer A (25 mM HEPES pH 7.5, 500 mM NaCl, 20 mM MgCl_2_ and 1 mM DTT) supplemented with 1 mg RNase A (Qiagen) per liter cells. The clear soluble fraction obtained after centrifugation was passed through amylose resin, washed with buffer B (50 mM HEPES pH 7.5, 500 mM NaCl, and 0.5 mM TCEP) in 0.5 M, 1 M, and 0.5 M NaCl gradient supplemented with 10 μg ml^−1^ RNase A, and eluted with buffer B supplemented with 40 mM maltose. The elution fractions were concentrated and treated with 1 mg ml^−1^ RNase A at 4 °C overnight, and separated by Hiload 16/60 Superdex 200 gel filtration chromatography (GE Healthcare) in buffer B. The dimer fractions were collected and concentrated. To obtain the monomer, A3H hap II dimer was subjected to RNase A (0.5 mg ml^−1^) treatment and Hiload 16/60 Superdex 75 gel filtration chromatography (GE Healthcare) in the presence of buffer B plus 1.5 M NaCl (1^st^), or 2 M NaCl (2^nd^), respectively, which resulted in monomeric species and released free RNAs from the dimers.

MBP-fused A3H m1 mutant dimer and monomer were purified with a protocol similar to that described above with modifications. The concentrated amylose elution fractions were subject to two rounds of 1 mg ml^−1^ RNase A treatment at 4 °C (RNase T1 at final concentration of 1 U μl^−1^ was also used for some batches) and Superdex 200 gel filtration chromatography in buffer B. In each round, the dimer fractions were collected and concentrated. The 260/280 ratio of the final dimer was between 0.92–1.0. To obtain MBP-fused A3H m1 monomer, the NaCl concentration of the dimer sample was adjusted to 1.5 M and the monomer fractions were collected after Superdex 75 gel filtration chromatography in buffer B with 1.5 M NaCl. The 260/280 ratio of the concentrated monomer was between 0.63–0.71.

The purification protocol of the cleaved A3H m1 W115A/C116S monomer for crystallization trials was similar to the previously published protocol^55^. There was no further RNase treatment needed after obtaining the amylose elution fractions. Superdex 75 gel filtration chromatography in buffer B was conducted to obtain the MBP-fused monomeric fractions. Then the MBP tag was cleaved with PreScission protease in buffer C (50 mM HEPES pH 7.5, 250 mM NaCl, 0.5 M arginine, 0.5 mM TCEP). The fractions containing the cleaved A3H monomer were obtained after Superdex 75 gel filtration chromatography in buffer C, collected, and concentrated for crystallization trials. The 260/280 ratio of the cleaved m1 W115A/C116S monomer was between 0.53–0.57. MBP-fused A3H monomer mutants (A3H m1 H114A and A3H m1 W115A/C116S) used in EMSA were purified with the same method described above without PreScission cleavage.

### Protein crystallization, data collection, structure determination and refinement

The cleaved monomeric A3H m1 W115A/C116S mutant protein was concentrated to about 4–7 mg ml^−1^ for crystallization screening. Initially crystals were obtained at 4 °C by sitting drop vapor-diffusion method in many conditions containing PEG (PEGs Suite, Qiagen). After optimization, crystals used for data collection were grown in 0.2 M Na thiocyanate and 4% PEG 20K at 4 °C. Diffraction data was collected in Advanced Photon Source 23-ID-D. Data sets were indexed, integrated and scaled using HKL2000 program package. The structure of A3H was determined by molecular replacement method by MOLREP (CCP4 suite) using the core structure of rA3G-CD1 (PDB ID: 5K81) with the loops being removed as the search model. The initial map was improved by NCS averaging and the model for the removed loops was build based on the improved map. The final structure was refined by PHENIX and manually checked in COOT. The statistics for diffraction data and structural determination/refinement is shown in Table 1.

### Electrophoretic mobility shift assay (EMSA)

Each 6-FAM labeled oligonucleotide at a specified concentration (1 nM 50 nt ssRNA, 10 nM 50 nt/13 nt/8 nt ssDNA) was titrated by MBP-fused A3H m1 dimer/monomer, A3H m1 H114A monomer, and A3H m1 W115A/C116S monomer up to 8 μM in 10 μl reaction volume containing 50 mM HEPES pH 7.5, 250 mM NaCl, 1 mM DTT, 2.5 mM EDTA and 10% glycerol. The reaction mixture was incubated on ice for 10 min and analyzed by 8% native PAGE. Typhoon RGB Biomolecular Imager (GE Healthcare) was used to visualize the images, ImageQuant TL (GE Healthcare) was used for image quantification, and GraphPad Prism software was used for curve fitting. Three independent experiments were performed.

### Cell culture and transfection for human cell-based assays

For studying the multimerization and subcellular localization of various A3H constructs in HEK293T cells (ATCC), A3H mutants generated in pcDNA 3.1(+) vector with an N-terminal FLAG tag were transfected into HEK293T cells. HEK293T cells were maintained in DMEM medium (Corning), supplemented with 10% FBS, 100 U ml^−1^ penicillin and 100 μg ml^−1^ streptomycin. Transfections of HEK293T cells were done by using X-tremeGENE 9 DNA Transfection Reagent (Roche) and following manufacturer’s recommendation. To detect the expression of various A3H constructs by Western blot, cell lysate samples were separated by SDS-PAGE, transferred onto PVDF membrane (EMD Millipore), and blotted with anti-FLAG M2 mAb (Sigma, 1:3000).

### Cell lysate fractionation analysis of A3H

Analysis of multimerization or HMW/LMW complex formation of A3H constructs in HEK293T cells was performed as previously described^55^, by fractionating cell lysate by SEC in FPLC. Briefly, at 72 h post-transfection, A3H-transfected HEK293T cells in 150 mm^2^ dishes were harvested, washed with PBS, and lysed in lysis buffer (50 mM HEPES pH 7.5, 125 mM NaCl, 0.6% NP-40 alternative, 0.5 mM TCEP, 10% glycerol final total volume was 1 ml after mixing with cells) with 1x Halt protease and phosphatase inhibitor (Thermo Fisher) for 15 min. After centrifugation and removing the surface lipid fraction, the clear supernatant fraction was loaded onto Superdex 200 10/300 GL column (GE Healthcare) equilibrated with 50 mM HEPES pH 7.5, 125 mM NaCl, 0.1% NP-40 alternative, 0.5 mM TCEP, 10% glycerol. Fractions were subjected to Western blot and deaminase assay. For RNase A treatment, the clear supernatant after lysis was incubated with 100 μg ml^−1^ RNase A on ice for 2 h before loading onto Superdex 200 10/300 GL column.

### Analysis of subcellular distribution of various A3H mutants

The subcellular distribution of various A3H mutants in HEK293T cells was analyzed by cell fractionation to separate the cytosol and nuclear fractions, followed by SDS-PAGE and Western blot analysis. At 48 h post-transfection, A3H-transfected HEK293T cells cultured in 6-well plates were harvested and washed with PBS. Cells were fractionated into whole cell, cytoplasmic and nuclear fractions by Nuclei EZ Prep kit (Sigma). The fractions were further treated with 2% SDS and benzonase (Sigma) to degrade chromosomal DNA. Subcellular fractions were analyzed by SDS-PAGE and Western blot using anti-FLAG M2 mAb to detect the various FLAG-A3H constructs. The FLAG-A3H band density was quantified with ImageJ software to determine the ratio of subcellular localization.

### Deaminase assay

At 48 h post-transfection, A3H-transfected 293T cells in 6-well plates were harvested, washed with PBS, and the whole cell lysates were prepared using M-PER protein extraction reagent (Thermo Fisher) with 1× Halt protease and phosphatase inhibitor. After centrifugation, the clear supernatant fraction was separated for deaminase assay. Prior to deaminase reaction, the total protein concentration was quantified by BCA protein assay kit (Pierce), the expression level of each A3H construct was quantified by Western blot, and normalized with the whole cell lysate transfected with the empty pcDNA 3.1(+) vector. Various concentration of A3H-transfected 293T cell lysates were incubated with 300 nM 5′−6-FAM-labeled 30 nt ssDNA substrates containing a target C or mC (5′-ATTTATATTATTTATT(m)CATATTTATATTTA-3′) in a final volume of 20 μl deaminase reaction mixture (25 mM HEPES, pH 7.0, 50 mM NaCl, 1 mM DTT, 0.1% Triton X-100, 0.1 mg ml^−1^ RNase A). The deaminase reaction was performed at 37 °C for 1 h, and then terminated by heat inactivation at 90 °C for 5 min. The bases of the deamination products U or T were subsequently cleaved by UDG (2.5 units, NEB) or TDG (0.5 μg, 3-fold excess amount of the complementary ssDNA was also added). The UDG reaction was performed at 37 °C for 1 h, and the TDG reaction was performed at 42 °C for overnight. The resulting abasic sites were hydrolyzed by 0.1 M NaOH at 90 °C for 10 min. The deamination products were separated on 20% urea denaturing gels, visualized by Molecular Imager FX (Bio-Rad) or Typhoon RGB Biomolecular Imager, and quantified by Quantity One 1-D Analysis Software (Bio-Rad) or ImageQuant TL. Deaminase activity (nM product/μg cell lysates) was determined as the product formation over enzyme concentration in an initial range where the product formation was linearly dependent on cell lysate concentration. Error bars were generated based on standard deviation of three independent data sets.

## Electronic supplementary material

Supplementary information

## References

[1] Conticello SG (2008). The AID/APOBEC family of nucleic acid mutators. Genome biology.

[2] Prochnow C, Bransteitter R, Chen XS (2009). APOBEC deaminases-mutases with defensive roles for immunity. *Science in China*. Series C, Life sciences/Chinese Academy of Sciences.

[3] Harris RS, Dudley JP (2015). APOBECs and virus restriction. Virology.

[4] Refsland EW, Harris RS (2013). The APOBEC3 family of retroelement restriction factors. Curr Top Microbiol Immunol.

[5] Tan L, Sarkis PT, Wang T, Tian C, Yu XF (2009). Sole copy of Z2-type human cytidine deaminase APOBEC3H has inhibitory activity against retrotransposons and HIV-1. FASEB J.

[6] OhAinle M, Kerns JA, Li MM, Malik HS, Emerman M (2008). Antiretroelement activity of APOBEC3H was lost twice in recent human evolution. Cell host & microbe.

[7] Desimmie BA (2014). Multiple APOBEC3 restriction factors for HIV-1 and one Vif to rule them all. J Mol Biol.

[8] Malim MH, Bieniasz PD (2012). HIV Restriction Factors and Mechanisms of Evasion. Cold Spring Harb Perspect Med.

[9] Feng Y, Goubran MH, Follack TB, Chelico L (2017). Deamination-independent restriction of LINE-1 retrotransposition by APOBEC3H. Sci Rep.

[10] Honjo T, Muramatsu M, Fagarasan S (2004). AID: how does it aid antibody diversity?. Immunity..

[11] Revy P (2000). Activation-induced cytidine deaminase (AID) deficiency causes the autosomal recessive form of the Hyper-IgM syndrome (HIGM2). Cell.

[12] Wakae K (2006). Evolution of class switch recombination function in fish activation-induced cytidine deaminase, AID. Int Immunol.

[13] Qiao Q (2017). AID Recognizes Structured DNA for Class Switch Recombination. Mol Cell.

[14] OhAinle M, Kerns JA, Malik HS, Emerman M (2006). Adaptive evolution and antiviral activity of the conserved mammalian cytidine deaminase APOBEC3H. Journal of Virology.

[15] LaRue RS (2008). The artiodactyl APOBEC3 innate immune repertoire shows evidence for a multi-functional domain organization that existed in the ancestor of placental mammals. BMC molecular biology.

[16] Aydin H, Taylor MW, Lee JE (2014). Structure-guided analysis of the human APOBEC3-HIV restrictome. Structure.

[17] LaRue RS (2009). Guidelines for naming nonprimate APOBEC3 genes and proteins. J Virol.

[18] Wang X (2011). Analysis of human APOBEC3H haplotypes and anti-human immunodeficiency virus type 1 activity. J Virol.

[19] Harari A, Ooms M, Mulder LC, Simon V (2009). Polymorphisms and splice variants influence the antiretroviral activity of human APOBEC3H. J Virol.

[20] Ooms M, Letko M, Binka M, Simon V (2013). The resistance of human APOBEC3H to HIV-1 NL4-3 molecular clone is determined by a single amino acid in Vif. PloS one.

[21] Nakashima M (2017). Mapping Region of Human Restriction Factor APOBEC3H Critical for Interaction with HIV-1 Vif. J Mol Biol.

[22] Li MM, Emerman M (2011). Polymorphism in human APOBEC3H affects a phenotype dominant for subcellular localization and antiviral activity. J Virol.

[23] Zhen A, Du J, Zhou X, Xiong Y, Yu XF (2012). Reduced APOBEC3H variant anti-viral activities are associated with altered RNA binding activities. PloS one.

[24] Mitra M (2015). Sequence and structural determinants of human APOBEC3H deaminase and anti-HIV-1 activities. Retrovirology.

[25] Ooms M, Majdak S, Seibert CW, Harari A, Simon V (2010). The localization of APOBEC3H variants in HIV-1 virions determines their antiviral activity. J Virol.

[26] Refsland EW (2014). Natural polymorphisms in human APOBEC3H and HIV-1 Vif combine in primary T lymphocytes to affect viral G-to-A mutation levels and infectivity. PLoS Genet.

[27] Feng Y (2015). Natural Polymorphisms and Oligomerization of Human APOBEC3H Contribute to Single-stranded DNA Scanning Ability. J Biol Chem.

[28] Dang Y (2008). Human cytidine deaminase APOBEC3H restricts HIV-1 replication. J Biol Chem.

[29] Gu J (2016). Biochemical Characterization of APOBEC3H Variants: Implications for Their HIV-1 Restriction Activity and mC Modification. J Mol Biol.

[30] Li J (2014). APOBEC3 multimerization correlates with HIV-1 packaging and restriction activity in living cells. J Mol Biol.

[31] Baig TT, Feng Y, Chelico L (2014). Determinants of efficient degradation of APOBEC3 restriction factors by HIV-1 Vif. J Virol.

[32] Chiu YL, Greene WC (2006). APOBEC3 cytidine deaminases: distinct antiviral actions along the retroviral life cycle. J Biol Chem.

[33] Bransteitter R, Pham P, Scharff MD, Goodman MF (2003). Activation-induced cytidine deaminase deaminates deoxycytidine on single-stranded DNA but requires the action of RNase. Proc. Natl. Acad. Sci. USA.

[34] Chelico L, Pham P, Calabrese P, Goodman MF (2006). APOBEC3G DNA deaminase acts processively 3′–>5′ on single-stranded DNA. Nat Struct Mol Biol.

[35] Starrett GJ (2016). The DNA cytosine deaminase APOBEC3H haplotype I likely contributes to breast and lung cancer mutagenesis. Nat Commun.

[36] Apolonia L (2015). Promiscuous RNA binding ensures effective encapsidation of APOBEC3 proteins by HIV-1. PLoS Pathog.

[37] York A, Kutluay SB, Errando M, Bieniasz PD (2016). The RNA Binding Specificity of Human APOBEC3 Proteins Resembles That of HIV-1 Nucleocapsid. PLoS Pathog.

[38] Wang T, Tian C, Zhang W, Sarkis PT, Yu XF (2008). Interaction with 7SL RNA but not with HIV-1 genomic RNA or P bodies is required for APOBEC3F virion packaging. J Mol Biol.

[39] Fu Y (2015). DNA cytosine and methylcytosine deamination by APOBEC3B: enhancing methylcytosine deamination by engineering APOBEC3B. Biochem J.

[40] Ito F, Fu Y, Kao SA, Yang H, Chen XS (2017). Family-Wide Comparative Analysis of Cytidine and Methylcytidine Deamination by Eleven Human APOBEC Proteins. J Mol Biol.

[41] Chen Q, Xiao X, Wolfe A, Chen XS (2016). The *in vitro* Biochemical Characterization of an HIV-1 Restriction Factor APOBEC3F: Importance of Loop 7 on Both CD1 and CD2 for DNA Binding and Deamination. J Mol Biol.

[42] Carpenter MA (2012). Methylcytosine and normal cytosine deamination by the foreign DNA restriction enzyme APOBEC3A. J Biol Chem.

[43] Nabel CS (2012). AID/APOBEC deaminases disfavor modified cytosines implicated in DNA demethylation. Nat Chem Biol.

[44] Popp C (2010). Genome-wide erasure of DNA methylation in mouse primordial germ cells is affected by AID deficiency. Nature.

[45] Bhutani N (2013). A critical role for AID in the initiation of reprogramming to induced pluripotent stem cells. FASEB J.

[46] Bhutani N (2010). Reprogramming towards pluripotency requires AID-dependent DNA demethylation. Nature.

[47] Kumar R (2013). AID stabilizes stem-cell phenotype by removing epigenetic memory of pluripotency genes. Nature.

[48] Kuong KJ, Loeb LA (2013). APOBEC3B mutagenesis in cancer. Nat Genet.

[49] Burns MB, Temiz NA, Harris RS (2013). Evidence for APOBEC3B mutagenesis in multiple human cancers. Nat Genet.

[50] Gwak M, Choi YJ, Yoo NJ, Lee S (2014). Expression of DNA cytosine deaminase APOBEC3 proteins, a potential source for producing mutations, in gastric, colorectal and prostate cancers. Tumori.

[51] Swanton C, McGranahan N, Starrett GJ, Harris RS (2015). APOBEC Enzymes: Mutagenic Fuel for Cancer Evolution and Heterogeneity. Cancer Discov.

[52] Roberts SA (2013). An APOBEC cytidine deaminase mutagenesis pattern is widespread in human cancers. Nat Genet.

[53] Prochnow C, Bransteitter R, Klein MG, Goodman MF, Chen XS (2007). The APOBEC-2 crystal structure and functional implications for the deaminase AID. Nature.

[54] Holden LG (2008). Crystal structure of the anti-viral APOBEC3G catalytic domain and functional implications. Nature.

[55] Xiao X (2017). Structural determinants of APOBEC3B non-catalytic domain for molecular assembly and catalytic regulation. Nucleic Acids Res.

[56] Xiao X, Li SX, Yang H, Chen XS (2016). Crystal structures of APOBEC3G N-domain alone and its complex with DNA. Nat Commun.

[57] Shandilya SM (2010). Crystal structure of the APOBEC3G catalytic domain reveals potential oligomerization interfaces. Structure.

[58] Kitamura S (2012). The APOBEC3C crystal structure and the interface for HIV-1 Vif binding. Nat Struct Mol Biol.

[59] Bohn MF (2013). Crystal structure of the DNA cytosine deaminase APOBEC3F: the catalytically active and HIV-1 Vif-binding domain. Structure.

[60] Byeon IJ (2013). NMR structure of human restriction factor APOBEC3A reveals substrate binding and enzyme specificity. Nat Commun.

[61] Siu KK, Sultana A, Azimi FC, Lee JE (2013). Structural determinants of HIV-1 Vif susceptibility and DNA binding in APOBEC3F. Nat Commun.

[62] Shi K, Carpenter MA, Kurahashi K, Harris RS, Aihara H (2015). Crystal Structure of the DNA Deaminase APOBEC3B Catalytic Domain. J Biol Chem.

[63] Bohn JA (2017). APOBEC3H structure reveals an unusual mechanism of interaction with duplex RNA. Nat Commun.

[64] Bohn MF (2015). The ssDNA Mutator APOBEC3A Is Regulated by Cooperative Dimerization. Structure.

[65] Lee, S. *et al*. Hydrogen bonds are a primary driving force for de novo protein folding of an APOBEC protein AID. *Acta Cryst*. **D73**, in press (2017). 10.1107/S2059798317015303 PMC5713874 29199976

[66] Conticello SG, Thomas CJ, Petersen-Mahrt SK, Neuberger MS (2005). Evolution of the AID/APOBEC family of polynucleotide (deoxy)cytidine deaminases. Mol Biol Evol.

[67] Shaban NM (2018). The Antiviral and Cancer Genomic DNA Deaminase APOBEC3H Is Regulated by an RNA-Mediated Dimerization Mechanism. Mol Cell.

[68] Lackey L, Law EK, Brown WL, Harris RS (2013). Subcellular localization of the APOBEC3 proteins during mitosis and implications for genomic DNA deamination. Cell Cycle.

[69] Suspene, R., Aynaud, M. M., Vartanian, J. P. & Wain-Hobson, S. Efficient deamination of 5-methylcytidine and 5-substituted cytidine residues in DNA by human APOBEC3A cytidine deaminase. *PloS one* **8** (2013). 10.1371/journal.pone.0063461 PMC3688686 23840298

[70] Fang Y, Xiao X, Li S, Wolfe A, Chen XS (2018). Molecular Interactions of an HIV-1 Restriction Factor APOBEC3F Catalytic Domain with a Single-Stranded DNA Substrate. Journal of Molecular Biology.

[71] Carpenter MA, Rajagurubandara E, Wijesinghe P, Bhagwat AS (2010). Determinants of sequence-specificity within human AID and APOBEC3G. DNA Repair (Amst).

[72] Rathore A (2013). The local dinucleotide preference of APOBEC3G can be altered from 5′-CC to 5′-TC by a single amino acid substitution. J Mol Biol.

[73] Chiu YL (2005). Cellular APOBEC3G restricts HIV-1 infection in resting CD4+T cells. Nature.

