# Supplementary material for: Understanding the Structure, Multimerization, Subcellular Localization and mC Selectivity of a Genomic Mutator and Anti-HIV Factor APOBEC3H
Source: Sci Rep. 2018 Feb 28;8:3763. doi: 10.1038/s41598-018-21955-0 (PMC5830531; doi:10.1038/s41598-018-21955-0)
Supplement: Supplementary file 1 — Supplementary information [file 41598_2018_21955_MOESM1_ESM.docx]

**Supplementary Information**

**Understanding the Structure, Multimerization, Subcellular Localization and mC Selectivity of a Genomic Mutator and Anti-HIV Factor APOBEC3H**

Fumiaki Ito^1, #^, Hanjing Yang^1, #^, Xiao Xiao^1, 2, 5^, Shu-Xing Li^1, 3^, Aaron Wolfe^1, 2^, Brett Zirkle^1, 2^, Vagan Arutiunian^1, 6^, Xiaojiang S. Chen^1, 2, 3, 4^ *

^1^ Molecular and Computational Biology, Departments of Biological Sciences and Chemistry; and ^2^ Genetic, Molecular and Cellular Biology Program, Keck School of Medicine; ^3^ Center of Excellence in NanoBiophysics; ^4^ Norris Comprehensive Cancer Center; University of Southern California, Los Angeles, CA 90089, USA.

^5^ Present address: Department of Infectious Diseases and Vaccines Research, Merck Research Laboratories, Merck & Co., Inc., West Point, PA, USA.

^6^ Present address: Department of Internal Medicine Meharry Medical College, Nashville, TN, USA.

^#^ These authors contributed equally to this work.

^*^To whom correspondence should be addressed.

Tel: +1 213 740-5487; FAX: +1 213 740 4340;

Email: xiaojiac@usc.edu

**Supplementary Table S1.** The deaminase activity and mC selectivity factor of loop 1 and loop 7 mutants of A3H.

| **A3H constructs** | | TCA  (nM product/μg cell lysate) | TmCA  (nM product/μg cell lysate) | Selectivity for mC  (mC/C) x 100 |
| --- | --- | --- | --- | --- |
| WT | | 171 ± 8 | 82.3 ± 4.8 | 48.3 |
| Loop-1 mutants | R17D | ND | ND | - |
|  | R18D | 141 ± 48 | 34.5 ± 2.1 | 24.5 |
|  | R20D | 105 ± 20 | 41.1 ± 8.3 | 39.0 |
|  | R21D | 8.7 | ND | - |
|  | R26D | 3.2 | ND | - |
|  | R18A/L19A | 387 ± 70 | 240 ± 32 | 62.0 |
|  | R21A | 490 ± 21 | 126 ± 4 | 25.7 |
|  | R26A | 110 ± 30 | 33.5 ± 19.1 | 30.5 |
|  | A28T | 702 ± 125 | 388 ± 69 | 55.3 |
| Loop-7 mutants | m1+W115A | 62.8 ± 0.9 | 21.4 ± 7.5 | 34.1 |
|  | m1 | 610 ± 202 | 471 ± 85 | 77.2 |
| Loop-swap  mutants | lp1_A3A | ND | ND | - |
|  | lp1_A3G-CD2 | ND | ND | - |
|  | lp7_A3A | 168 | 49 | 29.2 |
|  | lp7_A3G-CD2 | ND | ND | - |


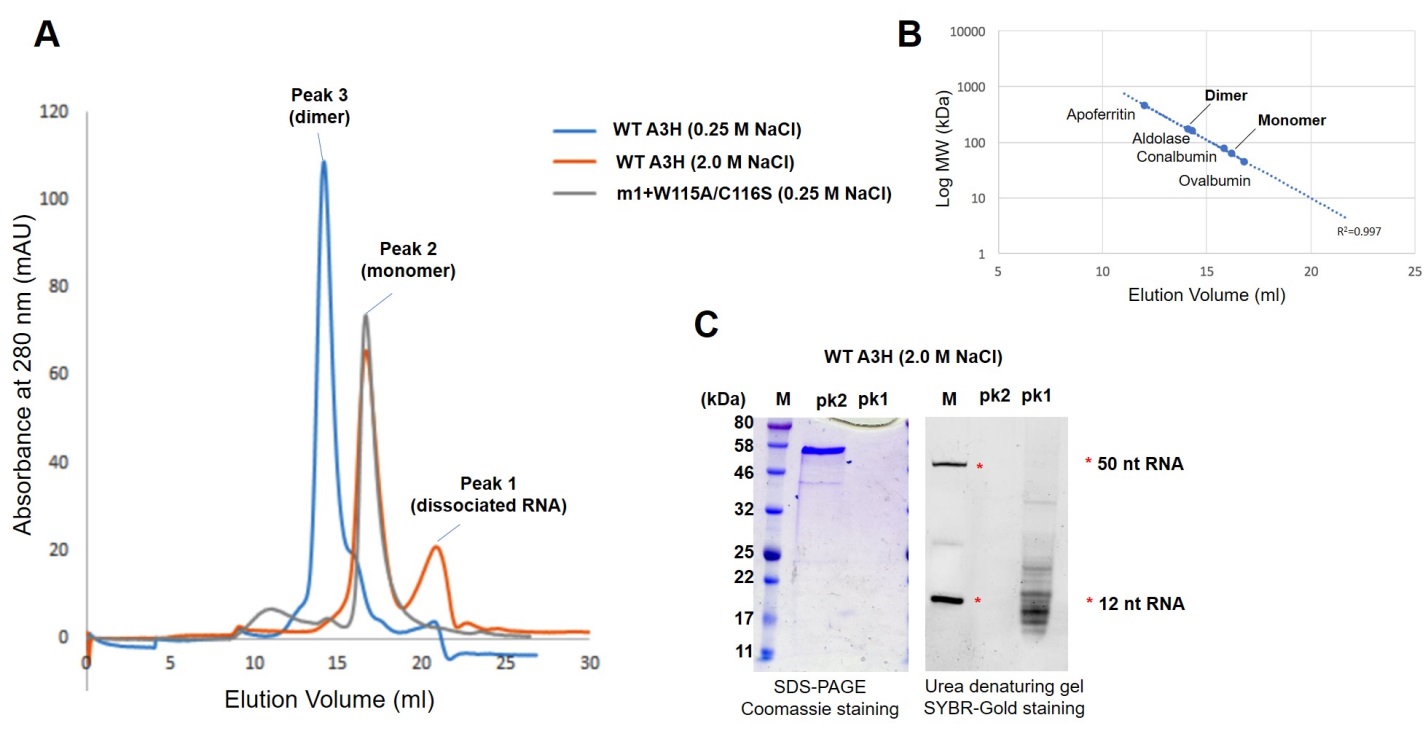


**Supplementary Figure S1.** Protein purification of wild-type A3H hap II. **(A)** SEC elution profiles of MBP-fusion of wild-type A3H dimeric and monomeric forms on Superdex 200. MBP-A3H forms a stable dimer after extensive RNase A treatment (blue). The purified dimer can dissociate to monomer and free RNA after RNase A treatment followed by 2.0 M or higher salt buffer (orange). The MBP-fusion of the monomeric mutant m1+W115A/C116S is used as monomeric size marker (grey). **(B)** The molecular weight standard curve for the SEC run in (A). **(C)** The SDS-PAGE gel (protein) and denaturing urea gel (nucleic acid) analysis of the peaks of the WT A3H treated with 2.0 M NaCl. The peak 1 (pk1) had a 260/280 ratio of 2.13, close to that of a typical RNA (the A260/280 for pure RNA is around 2.0, and pure DNA is 1.7).


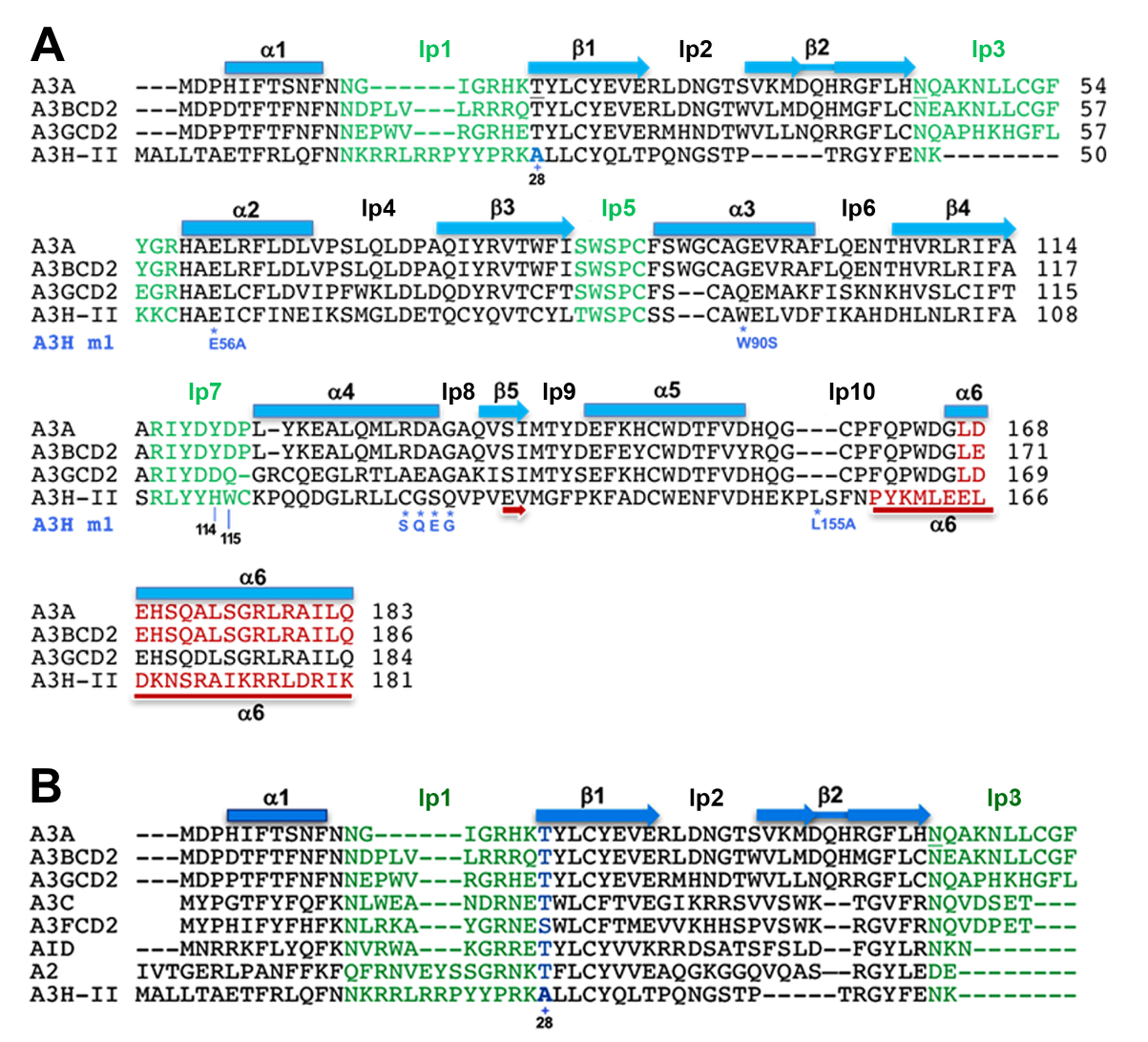


**Supplementary Figure S2.** Multiple sequence alignment of APOBEC proteins. **(A)** Sequence alignment of A3H haplotype II (A3H-II) with three other APOBECs. The nomenclature of secondary structures assigned on the top of the alignment is adopted based on those of APOBEC2 (Prochnow et al., Nature, 2007, 445:447-51), and the changes of secondary structures for A3H is indicated below the alignment. Mutations of the A3H m1 construct are shown.  **(B)** Multiple sequence alignment of the active APOBEC (except for A2) domains with known structures around loop 1 sequences, showing that the A28 residue unique to A3H is a T/S residue at the equivalent position in other APOBECs.


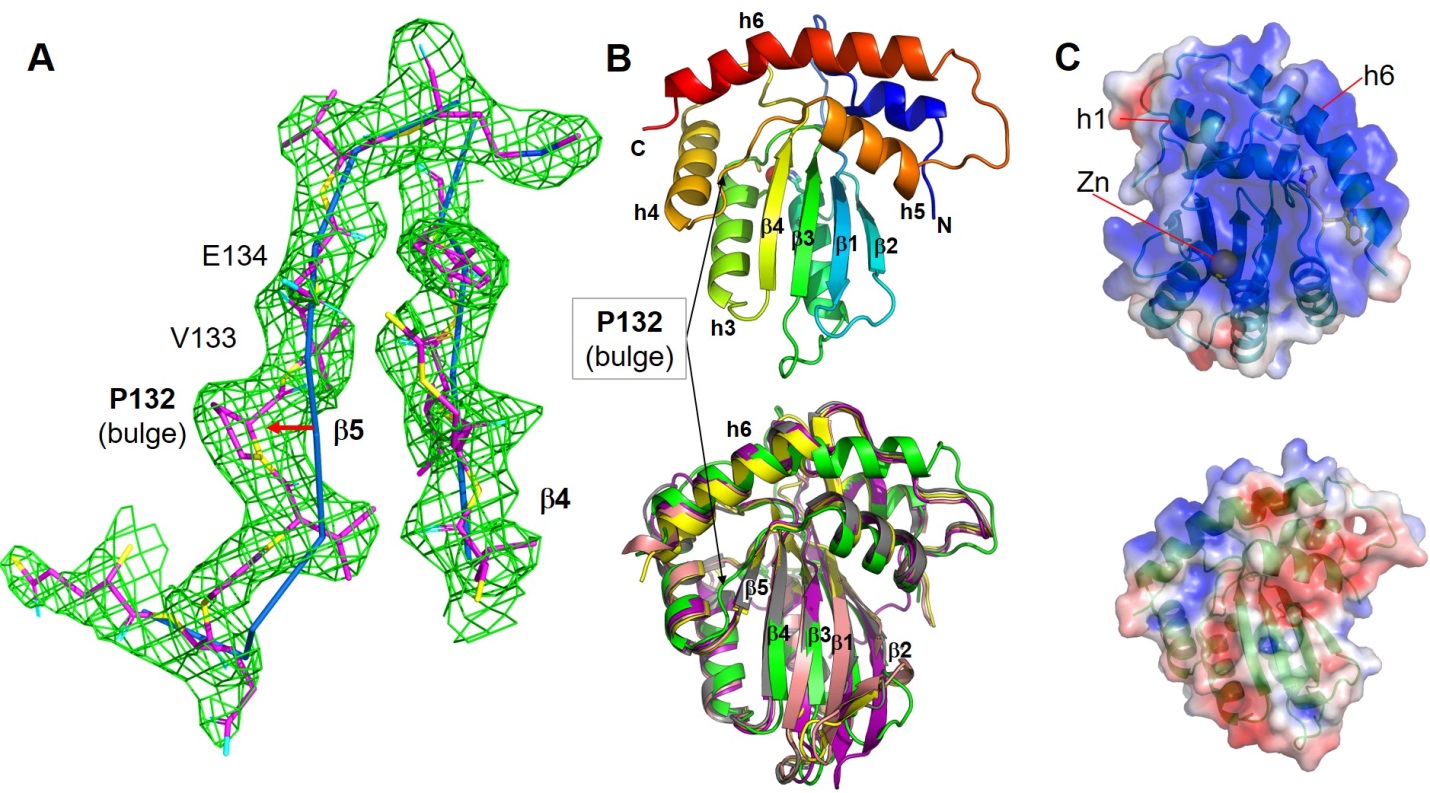


**Supplementary Figure S3.**Detailed structural features of A3H. **(A)** The electron density map for beta strand 4 (β4) and strand 5 (β5) of A3H monomeric structure in this report, showing P132 forming an outward-facing bulge away from β4, breaking the already short β5. The β5 strand in other the A3H dimer structure (5W3V) is shown in blue line. **(B)** The carton representation of A3H monomer structure (top), and the superimposition of A3H (green) with A3A (PDB: 4XXO, yellow), A3B-CD2 (PDB: 5CQI, salmon) and AID (PDB: 5W0R, purple), showing the bulge at residue P132 in the A3H monomer structure to break the short β5 strand. **(C)** The two opposite ends of A3H showing different charged features. The top panel is viewed from the Zn-center direction, and the bottom panel is the opposite end. The surface electrostatic potential colored according to calculated electrostatic potential of accessible surface area from -5 *kT/e* (red) to 5 *kT/e* (blue).

**
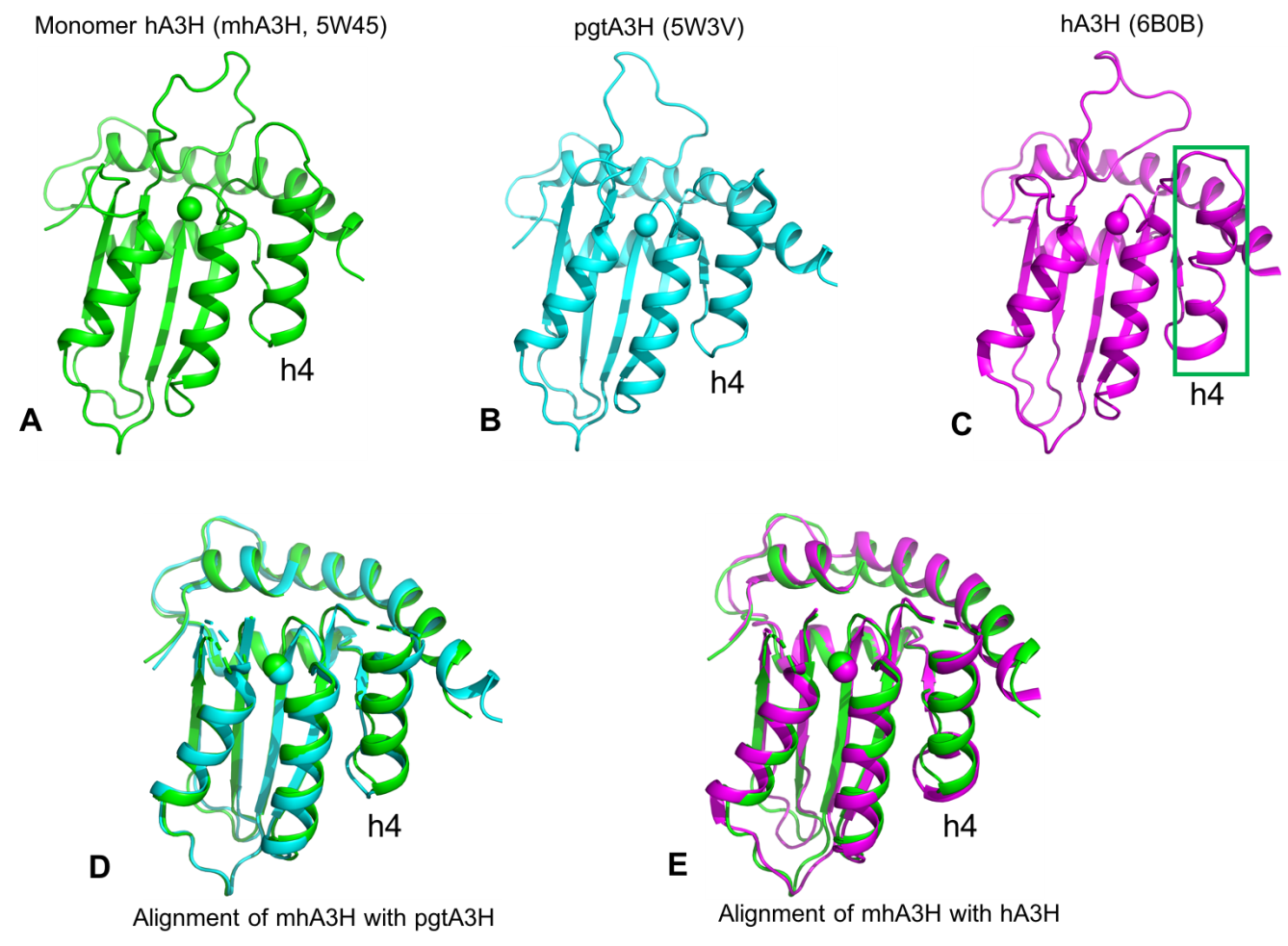
**

**Supplementary Figure S4.** The comparison of the A3H structure in this report (hA3H monomer, 5W45) with the other two published A3H structures, pgtA3H (5W3V, Nature Comm. 2017, Oct. 18) and human A3H (hA3H, (6B0B, Mol Cell, 2018, Jan 4). Our structure aligns very well with 5W3V (ptmA3H) (D). For clarity, the loops are deleted in the overlapping structures in D and E.


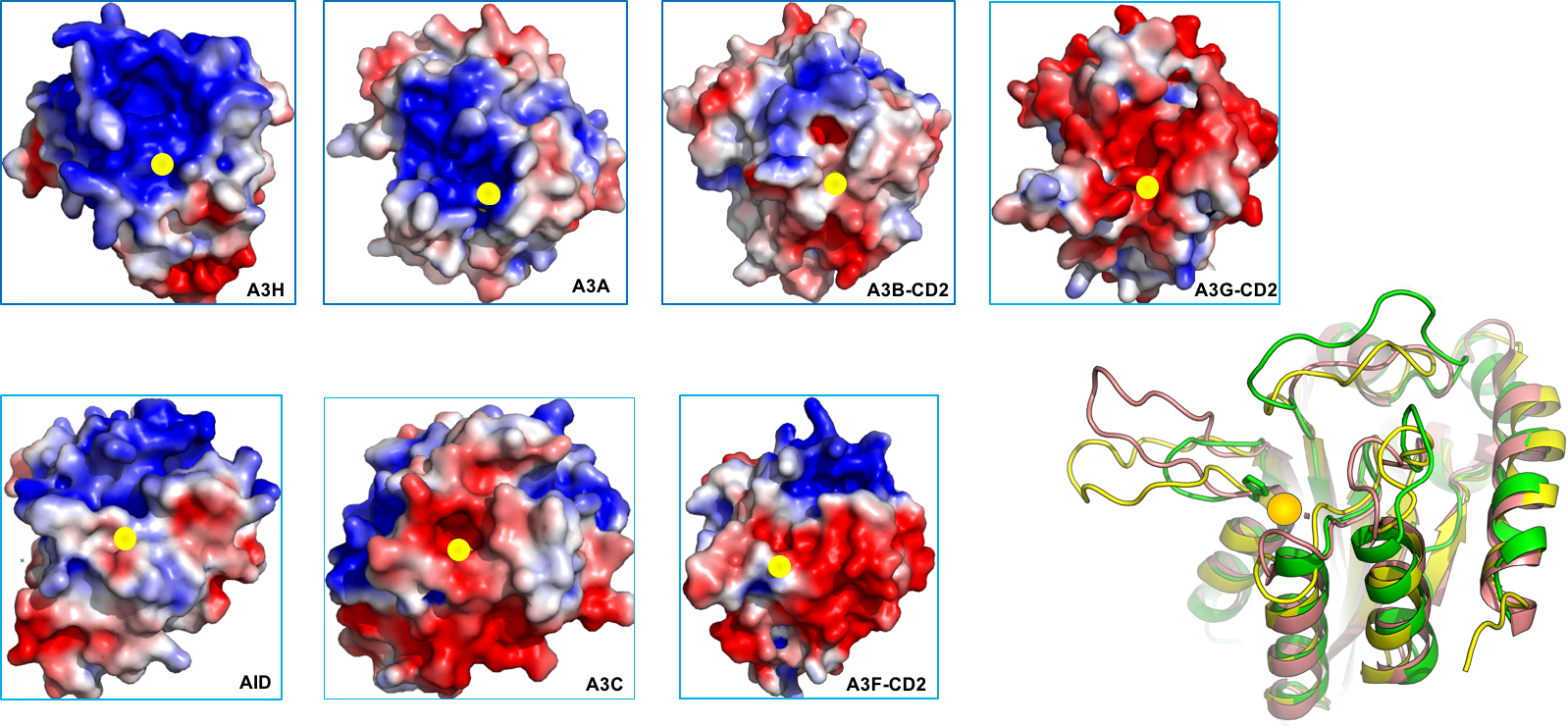


**Supplementary Figure S5.** The charged surface feature surrounding the Zn-active center of the active APOBEC domain structures, which reveal that the Zn-center of only A3H and A3A (PDB: 4XXO) are surrounded with positively charged areas. The Zn-center of the rest APOBECs shown here are surrounded by either neutral in A3B-CD2 (PDB: 5CQI), AID (PDB: 5W0R), A3F-CD2 (PDB: 3WUS) or even negatively charged areas in A3G-CD2 (PDB: 3IQS) and A3C (PDB: 3VOW). The ribbon diagram on the right shows the general orientation of views for the surface figures.


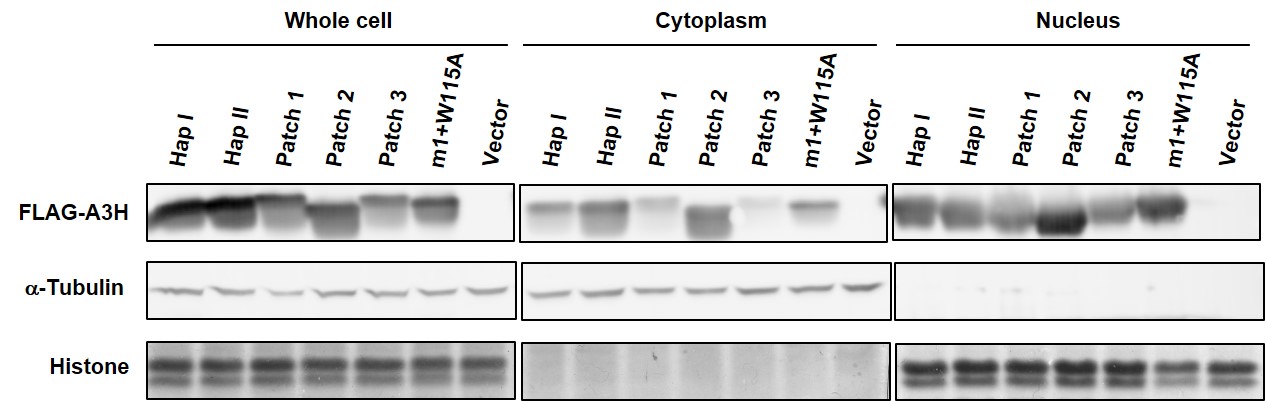


**Supplementary Figure S6.** Cell fractionation analysis of A3H and various mutants, showing the

distribution between nucleus and cytosol in HEK293T cells. Transfected 293T cells expressing

wild-type A3H hap I (mostly nuclear), A3H hap II, and various hap II mutants were fractionated

into whole cell, cytoplasmic and nuclear fractions. FLAG-A3H proteins, as well as α-tubulin

(cytoplasm only) and Histones (nucleus only) in each fraction were analyzed by Western blot.

**Below are original gel images shown in the Figures for the paper**

B


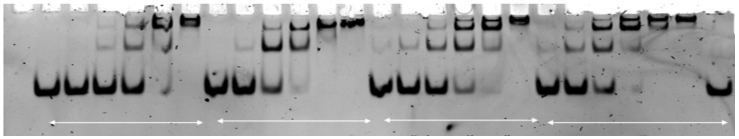


C


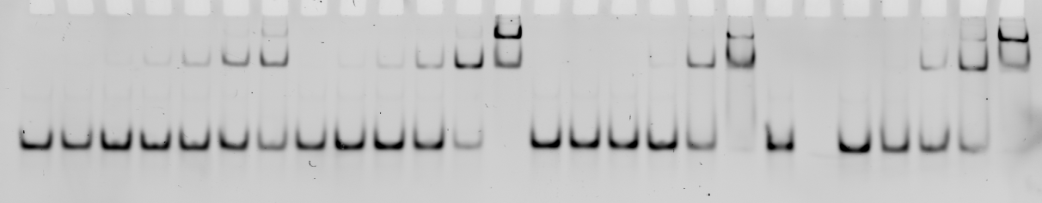


D


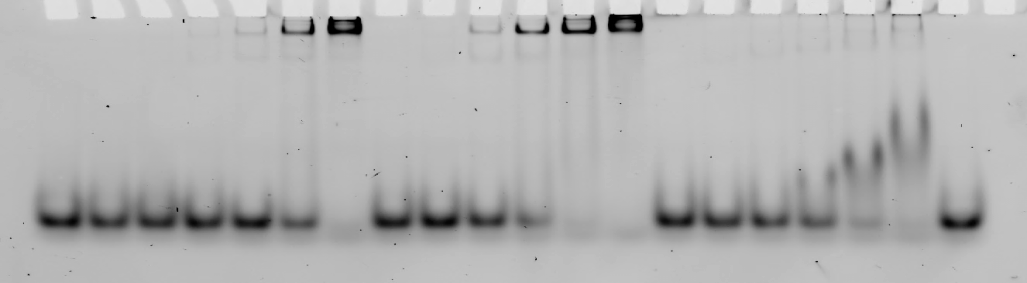


E


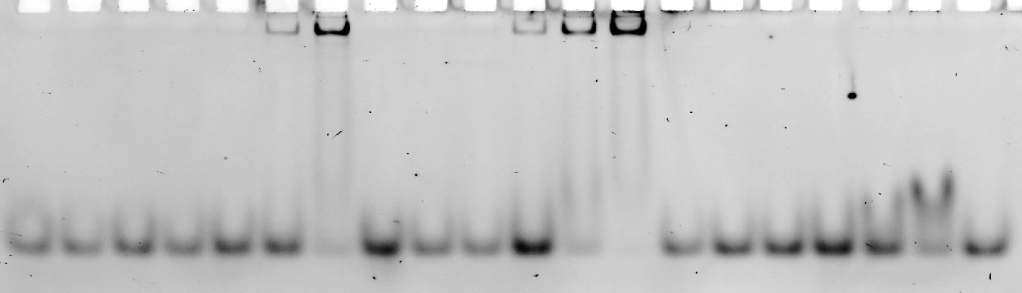


Original uncropped gels for Figure. 2B-E (ssDNA and ssRNA binding assay of A3H mutants).


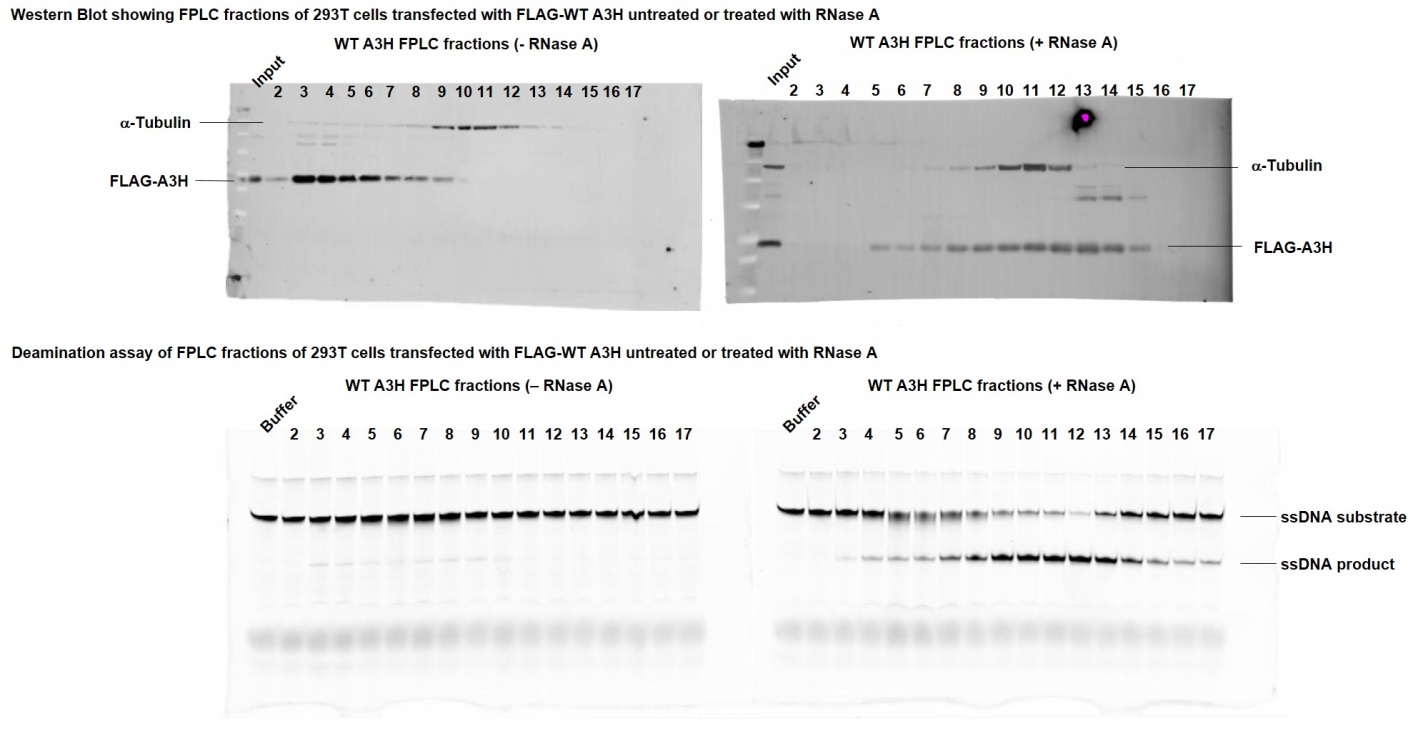


Original uncropped gels for Figure. 3 (HMW and LMW formation in HEK293T cells without or with RNase A treatment).


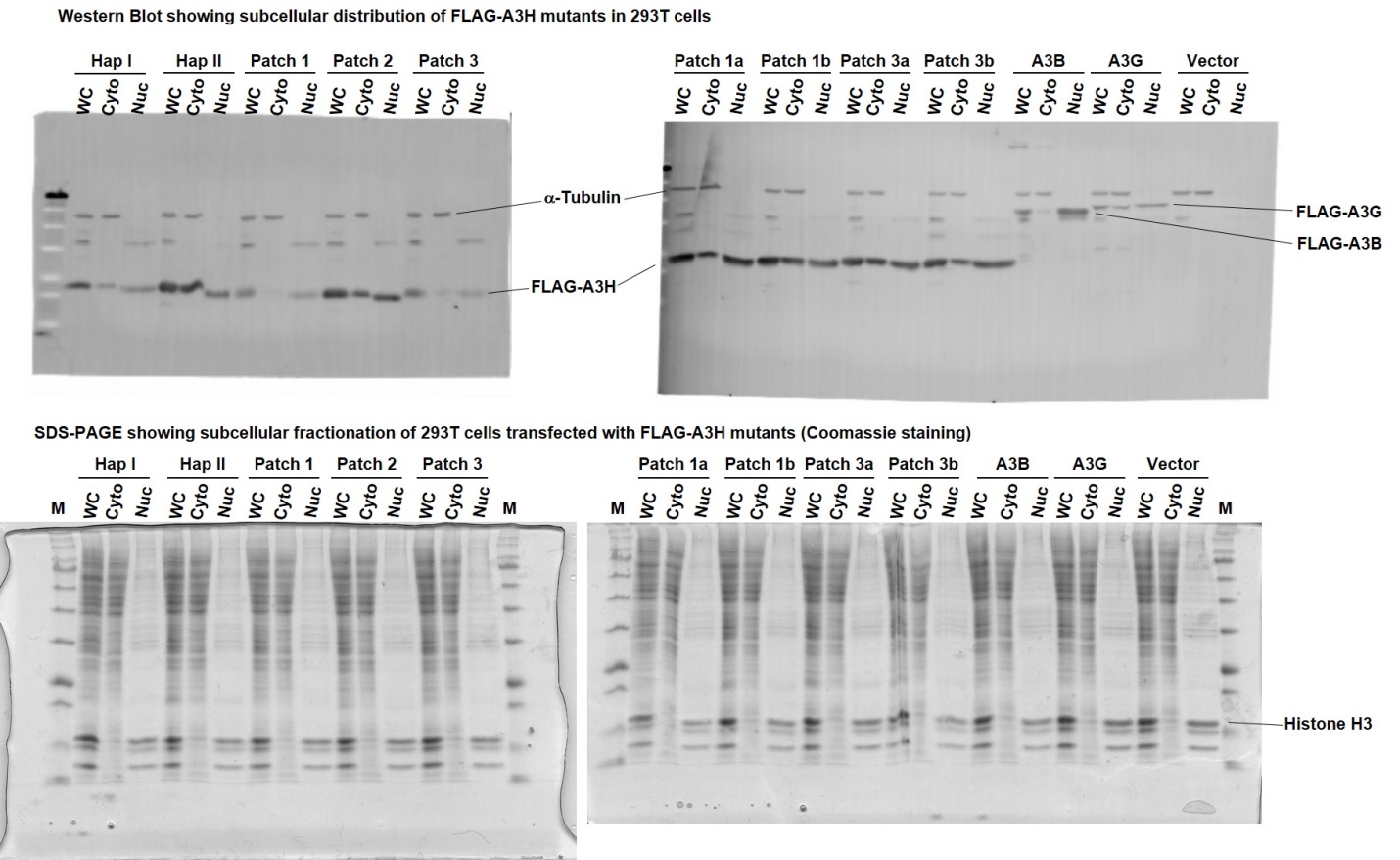


Original uncropped gels for Figure. 4B (subcellular localization of A3H patch mutants).


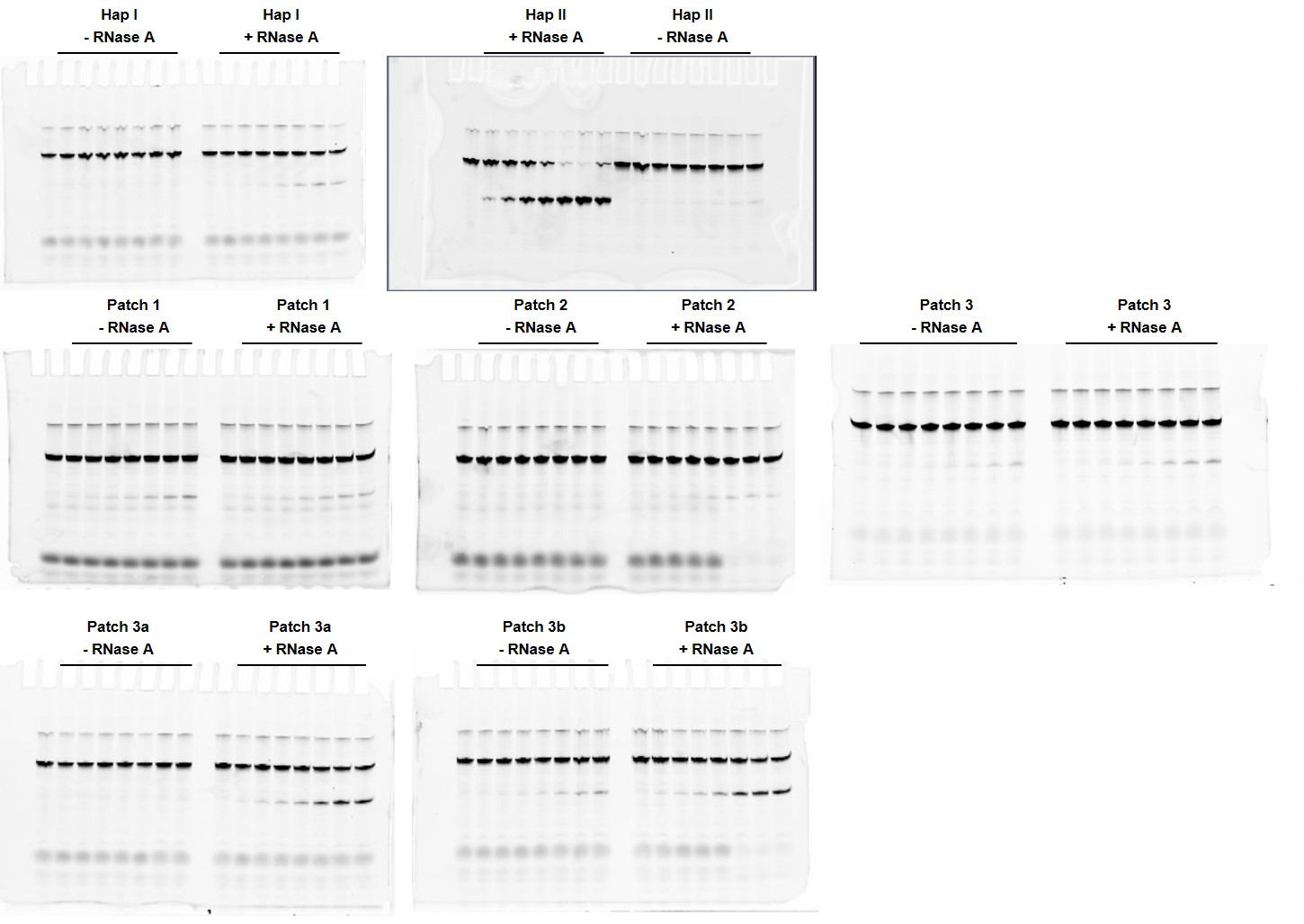


Original uncropped gels for Figure. 4C (deaminase assay of A3H patch mutants with/without RNase A).


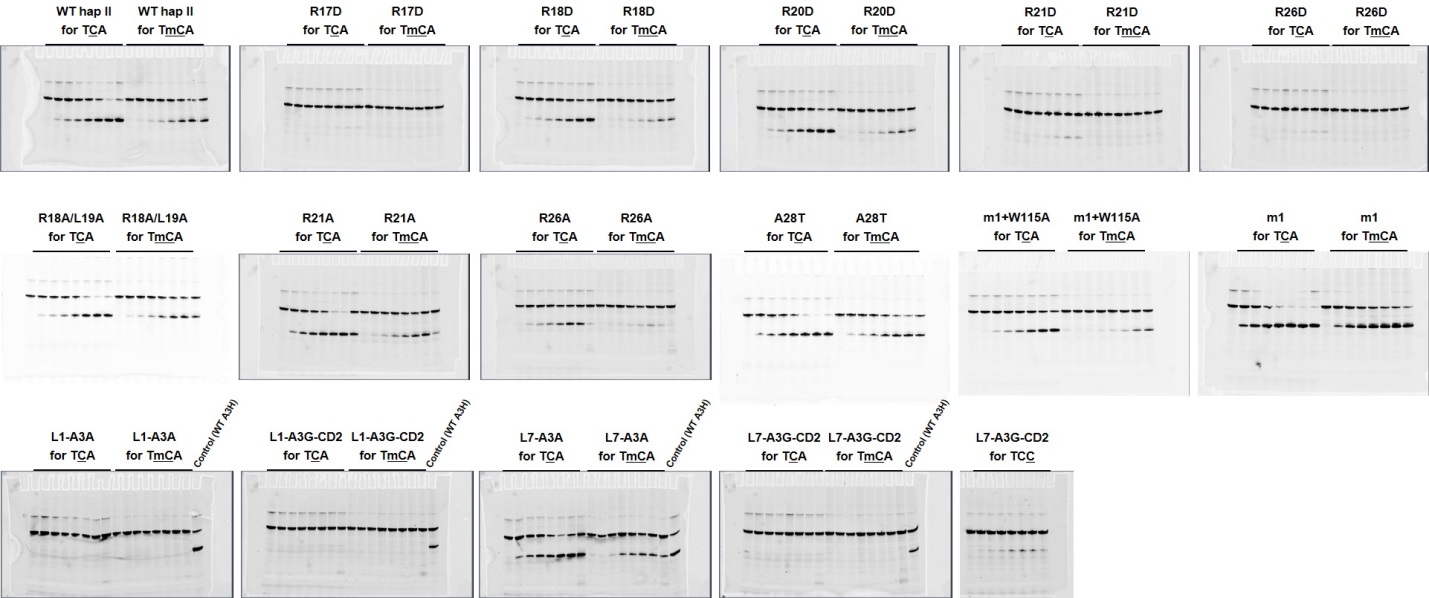


Original uncropped gels for Figure. 6A-D (deaminase assay of A3H mutants for -TCA- and -TmCA- containing ssDNA substrate).


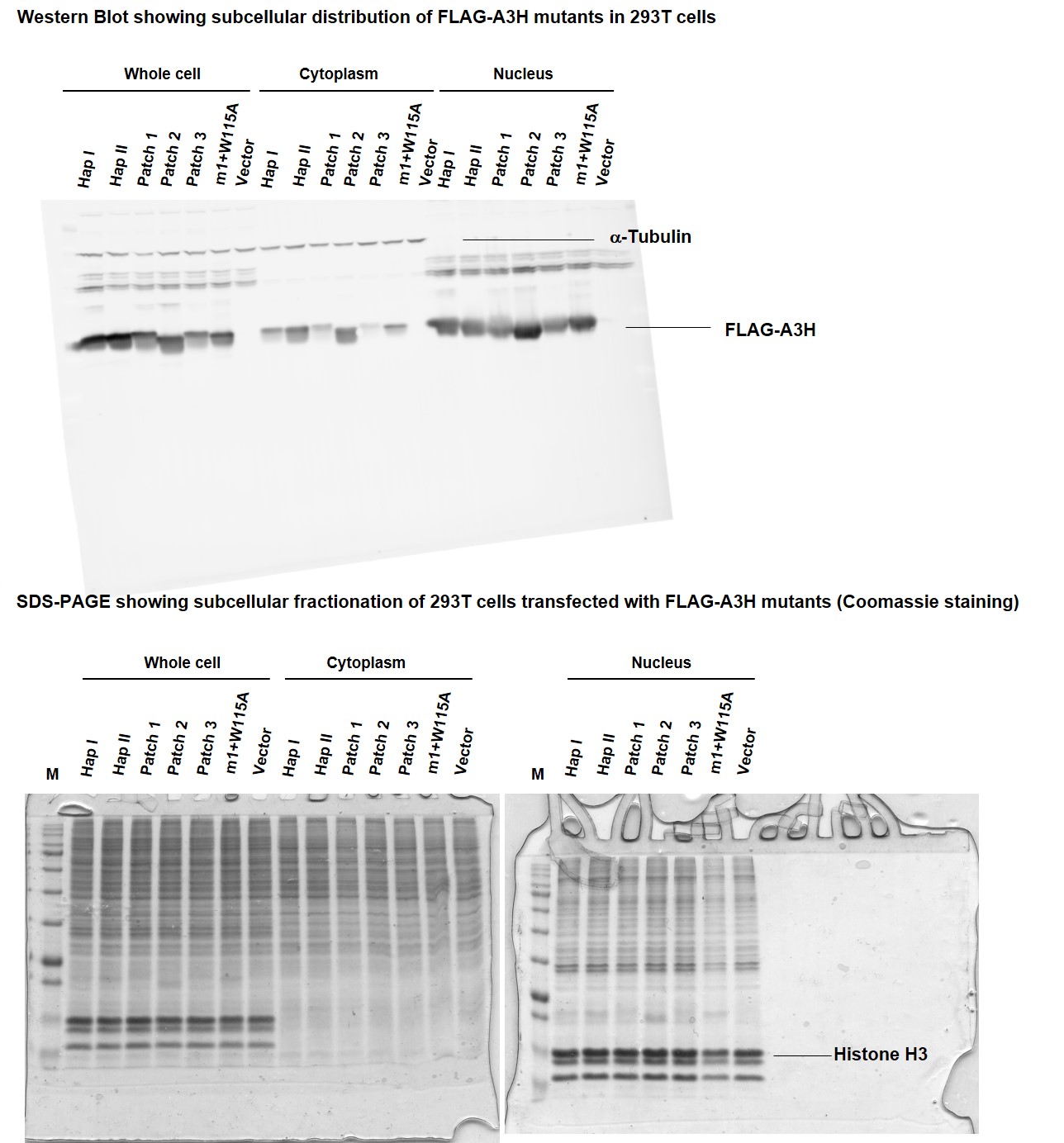


Original uncropped gels for Supplementary Figure. S6 (subcellular localization of A3H patch mutants).
